# Pulmonary Drug Delivery in the Era of Nanomedicine: From Biological Barriers to Artificial Intelligence-Driven Optimization

**DOI:** 10.3390/ph19071095

**Published:** 2026-07-16

**Authors:** Ibrahim A. Alradwan, Sarah A. Allabban, Aram S. Aleissa, Norah M. Alqahtani, Hamzah A. Alghamdi, Nojoud Al Fayez, Manal A. Alshabibi, Essam A. Tawfik, Fahad A. Almughem, Abdullah A. Alshehri

**Affiliations:** Advanced Diagnostics and Therapeutics Institute, Health Sector, King Abdulaziz City for Science and Technology (KACST), Riyadh 11442, Saudi Arabia

**Keywords:** pulmonary drug delivery, in silico modeling, inhalation, nanoparticle, artificial intelligence (AI)

## Abstract

Pulmonary drug delivery has become a vital route for both local and systemic treatments because of the unique structure and function of the respiratory system. Unlike oral and injectable dosage forms, inhalation offers a non-invasive, direct route to deliver medicines to the lungs, bypassing gastric degradation and first-pass hepatic metabolism. Common forms such as aerosols, solutions, suspensions, and dry powders are frequently used to treat respiratory diseases like asthma and chronic obstructive pulmonary disease (COPD). However, their effectiveness is often limited by physiological and biopharmaceutical barriers, such as mucociliary clearance, enzymatic degradation, and nonspecific deposition, which reduce drug retention and bioavailability. These issues are especially critical for poorly soluble or sensitive molecules, leading to lower drug concentrations at the target site and necessitating frequent dosing. To address these challenges, advanced nanoparticle-based delivery systems are being developed to improve drug stability, targeting, and controlled release within the lungs. At the same time, computational methods, including deposition modeling, physiologically based pharmacokinetic (PBPK) simulations, and AI-driven optimization, are increasingly used in formulation development to predict in vivo performance and boost translational success. This review covers the physiological and biological barriers to pulmonary drug delivery, explores major inhalation routes and dosage forms, and discusses new therapeutic strategies and nanoparticle platforms. It also highlights the growing role of in silico modeling and AI in accelerating the design and optimization of pulmonary treatments, while addressing current challenges, limitations, and regulatory issues in translating pulmonary nanomedicine into clinical practice.

## 1. Introduction

Pulmonary drug delivery has gained substantial attention as a clinically valuable route for both local and systemic therapy due to the unique structural and physiological properties of the respiratory tract. In contrast to oral and parenteral administration, inhalation enables direct delivery of therapeutics to the lung through a non-invasive route that bypasses gastrointestinal degradation and hepatic first-pass metabolism [1,2]. These characteristics can support rapid absorption, fast pharmacological onset, and improved dose efficiency, making the pulmonary route attractive for respiratory diseases as well as selected systemic applications requiring rapid bioavailability [1,2]. Also, the lung provides a favorable physiological interface for drug absorption due to its large surface area, estimated at 70 to 100 m^2^ in adults; the extremely thin alveolar capillary barrier of approximately 0.5 to 1 µm; dense vascularization; and relatively low enzymatic activity compared with the gastrointestinal tract [2]. Together, these features facilitate efficient drug exchange with the systemic circulation and make pulmonary administration especially attractive for labile therapeutics, including peptides, proteins, macromolecules, and vaccines, which are often degraded or poorly absorbed after oral delivery [1,2].

Conventional pulmonary dosage forms, including aerosols, solutions, suspensions, and dry powders, have long been used in clinical practice, particularly for the management of asthma and chronic obstructive pulmonary disease (COPD) [3]. Despite their therapeutic utility, these systems remain limited by physiological and biopharmaceutical barriers that reduce pulmonary residence time and drug availability, including airway clearance mechanisms, enzymatic degradation, and non-specific deposition [4]. These limitations are particularly challenging for poorly soluble or biologically fragile agents, which may fail to achieve adequate and sustained drug concentrations within diseased lung tissue, thereby requiring frequent dosing and compromising therapeutic outcomes [5]. In many pulmonary disorders, including lower respiratory tract infections, pulmonary fibrosis, tuberculosis, and lung cancer, the effectiveness of inhaled therapy is further reduced by disease-associated changes in airway structure, mucus properties, and local immune responses [1]. These pathological alterations can impair drug deposition, penetration, and retention, thereby creating a strong rationale for advanced delivery systems capable of improving therapeutic localization and persistence within the lung microenvironment [6].

Among these approaches, nanotechnology has emerged as a versatile platform for pulmonary drug delivery, enabling the design of carriers with novel physicochemical properties and broad cargo compatibility. Nanoparticle-based systems can encapsulate diverse therapeutic agents, ranging from small molecules to nucleic acids, while improving drug stability, enhancing apparent solubility, and supporting controlled release and cellular uptake [7]. As a result, nanocarriers are increasingly being explored as a means to improve the efficiency and precision of pulmonary therapeutics [8]. Despite these advantages, the clinical translation of pulmonary nanomedicine remains constrained by significant biological, technological, and regulatory challenges, which hinder formulation stability, aerosol performance, scalable manufacturing, and long-term safety [9]. Endogenous lung defense mechanisms, formulation instability during aerosolization and storage, difficulties in achieving reproducible manufacturing, device compatibility constraints, and unresolved questions regarding the long-term safety of repeated inhalation of nanomaterials continue to hinder clinical progress [10]. Furthermore, effective pulmonary administration necessitates meticulous regulation of aerodynamic characteristics and subsequent particle distribution. Consequently, nanoparticles are often engineered into porous nano-in-micro carriers, which enable efficient deposition within the lower respiratory tract while preserving the functional advantages of the nanoscale payload after deposition [11,12]. Surface engineering strategies, including polymer coating and ligand conjugation, can further reduce mucociliary entrapment, limit macrophage-mediated clearance, and improve transport across mucus barriers, which is particularly important in diseases such as lung cancer and pulmonary fibrosis, where localized and cell-selective delivery may improve efficacy while reducing systemic toxicity [13,14]. Consistent with these challenges, only a small number of inhaled nanoparticle formulations have advanced beyond the preclinical stage. One notable example is liposomal amikacin (Arikayce^®^), approved for the treatment of nontuberculous mycobacterial lung disease, which provides proof of concept for the translational potential of pulmonary nanomedicine [6]. 

Despite recent advances, clinical, technological, and translational obstacles persist, limiting the full potential and clinical translation of pulmonary drug delivery. These challenges are further compounded by the need to align therapeutic modalities with appropriate inhalation dosage forms and devices, optimize nanoparticle design for diverse cargo types, including small molecules, proteins, nucleic acids, vaccines, and gene-editing systems, and address scale-up, quality control, device-formulation compatibility, and regulatory requirements. Overcoming these limitations will require an integrated framework that combines advanced nanocarrier engineering with mechanistic and data-driven in silico approaches, including deposition modeling, physiologically based pharmacokinetic (PBPK) simulations, and artificial intelligence (AI)-driven optimization, to improve formulation development, predict in vivo performance, and support more precise and translatable pulmonary therapies.

This review provides an integrated translational perspective on pulmonary drug delivery. It examines the biological barriers associated with inhalation, evaluates current clinical dosage forms, and critically assesses emerging nanoplatforms across diverse therapeutic modalities. It also highlights the role of computational modeling and AI in understanding formulation-device interactions and in advancing next-generation inhaled therapeutics by linking biological barriers, dosage forms, devices, therapeutic modalities, nanoparticle design, and in silico optimization within a single framework for formulation design and clinical translation.

## 2. Lung Physiological and Biological Barriers to Drug Delivery

**Figure 1:**
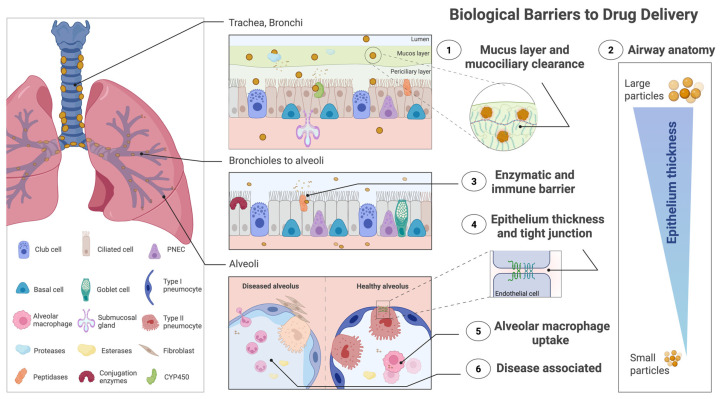
Major biological barriers to pulmonary drug delivery include airway anatomy, mucus and mucociliary clearance, epithelial tight junctions, enzymatic and immune barriers, alveolar macrophage uptake, and disease-associated alterations. Created in BioRender. AlFayez, N. (2026). https://BioRender.com/u6f7wl9 (accessed on 10 July 2026).

The lung is an attractive route for drug administration. Still, its therapeutic potential is constrained by a series of physiological, physicochemical, cellular, and immunological barriers that govern particle deposition, residence time, epithelial transport, and clearance (Figure 1). These barriers are not passive obstacles; rather, they are dynamic determinants of local and systemic drug exposure and therefore must be considered in the rational design of inhaled therapeutics. A mechanistic understanding of pulmonary physiological and barrier function is thus essential for optimizing formulation performance and translational success.

The respiratory tract is broadly divided into the conducting and respiratory zones, each with distinct implications for inhaled drug delivery. The conducting zone extends from the nasal cavity to the terminal bronchioles and primarily functions in air conduction, filtration, humidification, and warming. In contrast, the respiratory zone, comprising the respiratory bronchioles, alveolar ducts, and alveolar sacs, is specialized for gas exchange and serves as the principal site for systemic drug absorption following inhalation [15,16]. Importantly, the epithelial structure exhibits gradual alterations across this progression. The conducting airways are lined with ciliated columnar epithelium containing goblet cells and, in the larger airways, submucosal glands. In contrast, the distal lung transitions to a thinner, minimally ciliated epithelium that is optimized for gas exchange rather than defense [17]. As regional deposition directly influences clearance mechanisms, absorption efficiency, and access to target cells, the specific anatomical site of drug deposition is a critical determinant of therapeutic efficacy [15].

The alveolar epithelium forms a highly specialized, selectively permeable barrier that supports rapid gas exchange while restricting the uncontrolled entry of exogenous materials. It is composed primarily of type I pneumocytes, which cover most of the alveolar surface and mediate exchange, and type II pneumocytes, which produce surfactant and contribute to epithelial repair and homeostasis [18]. Tight junctions between epithelial cells preserve barrier integrity and limit paracellular transport, particularly for hydrophilic and high-molecular-weight (HMW) agents such as peptides and proteins. As a result, transport across the alveolar barrier is strongly influenced by the physicochemical properties of the therapeutic, with lipophilic compounds tending to favor transcellular transport, whereas hydrophilic molecules face greater diffusional restriction [19]. These features are particularly relevant for advanced inhaled therapeutics, including biologics and nanocarriers, whose pulmonary absorption depends on both barrier compatibility and regional deposition [19].

In the conducting airways, the mucus layer and mucociliary escalator constitute a major front-line defense against inhaled particulates and pathogens. Airway mucus, which is rich in mucins, exhibits viscoelastic properties that facilitate the trapping of foreign materials, including inhaled therapeutics [20,21]. Coordinated ciliary beating then drives mucus transport toward the pharynx, thereby reducing drug residence time and limiting opportunities for epithelial interaction and absorption [20,21]. This clearance system is especially relevant to inhaled formulations intended for prolonged airway retention, as interactions with mucus can markedly reduce the effective dose delivered to distal lung regions. Accordingly, therapeutic performance in the airways depends not only on drug potency but also on the formulation’s ability to avoid premature mucus entrapment and on clearance, which is influenced by particle size, as larger particles exhibit higher retention within the mucus layer and are more susceptible to macrophage uptake [6].

In the distal lung, cellular clearance is performed by alveolar macrophages, which serve as key sentinels of innate immunity by recognizing and engulfing deposited foreign materials, including therapeutic particles [22]. Following phagocytosis, inhaled materials may undergo lysosomal degradation, lymphatic transport, or secondary clearance through mucociliary mechanisms [22]. Additional immune cell populations, including neutrophils, dendritic cells, and lymphocytes, can further amplify recognition and inflammatory signaling in response to inhaled materials [23]. In pulmonary drug delivery, these processes may reduce drug retention and bioavailability, particularly in particulate systems, and may also influence local tolerability. Immune recognition is not only a safety concern but also a central determinant of formulation persistence and therapeutic performance in the alveolar region. In addition, pulmonary epithelial cells express multiple drug-metabolizing enzymes, including cytochrome P450 isoforms, esterases, proteases, peptidases, and conjugating enzymes, all of which can contribute to the biotransformation or degradation of inhaled drugs [24]. Oxidative metabolism, hydrolysis, peptide bond cleavage, and subsequent conjugation may collectively reduce the stability, bioavailability, and pharmacological activity of delivered agents [24,25]. These barriers are particularly important for labile cargos such as peptides, proteins, and other biologically derived therapeutics, for which enzymatic degradation may substantially limit effective pulmonary exposure. Thus, the metabolic environment of the lung must be considered alongside deposition and clearance when designing advanced inhaled formulations [24,25].

One of the major factors complicating pulmonary drug delivery is disease-driven alteration of lung structure and barrier function. Deposition patterns are influenced by breathing behavior, airway geometry, and upper airway anatomy, all of which may be significantly modified under pathological conditions [26]. In addition, inflammatory, infectious, obstructive, and fibrotic lung diseases can induce epithelial injury, mucus hypersecretion, airway remodeling, immune cell infiltration, and altered macrophage activity, thereby changing barrier integrity, regional deposition, permeability, and clearance kinetics [27]. These pathological changes can reduce drug penetration, shorten residence time, and introduce substantial variability in therapeutic response, particularly in diseased lungs [6,27,28]. These considerations underscore the need for disease-aware, patient-informed optimization of inhaled therapeutics rather than relying on a one-size-fits-all formulation strategy.

It should be noted that these physiological barriers are not static but undergo disease-specific alterations that significantly influence the performance of inhaled formulations. In asthma and COPD, airway narrowing, mucus hypersecretion, and heterogeneous ventilation reduce peripheral deposition and increase proximal airway retention, highlighting the need for aerosols with optimized aerodynamic diameters and prolonged airway residence [29]. In cystic fibrosis (CF), highly viscoelastic and dehydrated mucus severely restricts particle diffusion, necessitating mucus-penetrating nanocarriers with hydrophilic or PEGylated surfaces to minimize adhesive interactions with mucin networks [30]. Pulmonary infections, including tuberculosis, involve extensive macrophage activation and granuloma formation, making macrophage-targeted nanoparticles advantageous for intracellular drug delivery, while also necessitating strategies to overcome enhanced phagocytic clearance [31]. In idiopathic pulmonary fibrosis (IPF), extracellular matrix deposition, epithelial remodeling, and reduced alveolar accessibility limit tissue penetration, preferring ultrasmall nanoparticles or systems capable of enhanced interstitial transport. Conversely, lung cancer presents highly heterogeneous tumor architecture, abnormal vasculature, elevated interstitial pressure, and an immunosuppressive microenvironment, requiring nanocarriers engineered for prolonged retention, tumor-specific targeting, and controlled intracellular drug release [32]. These disease-dependent alterations emphasize that successful pulmonary drug delivery requires formulation strategies tailored not only to the physicochemical properties of the therapeutic agent but also to the pathological characteristics of the target lung disease.

## 3. Pulmonary Drug Delivery Routes and Dosage Forms

The pulmonary route of administration is a flexible platform for both local and systemic drug delivery. Local delivery is especially important in treating asthma, COPD, and pulmonary infections because it allows high drug concentrations at the site of action while reducing systemic exposure [33]. Conversely, systemic delivery via the lung takes advantage of the large absorptive surface, thin alveolar membrane, and dense blood supply of the distal lung, which together allow for rapid drug absorption and a quick onset of action, as shown clinically with inhaled insulin formulations like Afrezza^®^ [34,35]. These properties make inhalation especially attractive for therapeutics that benefit from non-invasive delivery, rapid pharmacokinetics, or reduced first-pass metabolism. In this context, nanoparticle-based formulations offer additional advantages by enhancing drug solubility and stability, boosting penetration through pulmonary barriers, prolonging lung residence time, and enabling controlled or targeted delivery [10,36].

Achieving these benefits in practice depends on effective aerosol generation, as pulmonary formulations must first be converted into stable, respirable aerosols to deliver drugs efficiently to the lungs.

### 3.1. Aerosolization Strategies

Aerosolization is a key factor in pulmonary drug delivery because it determines whether a formulation can be turned into a respirable aerosol and reproducibly delivered to the target lung area [37]. Effective inhalation systems must produce particles or droplets within an aerodynamic range suitable for deposition in the lower airways while maintaining formulation integrity during aerosolization [38]. This requirement is especially crucial for biologics and nanocarriers, which can undergo aggregation, premature drug release, or loss of bioactivity under aerosolization stress. For example, nebulizing coated poly(lactic-co-glycolic acid) (PLGA) nanoparticles with a commercial Aeroneb^®^ vibrating-mesh nebulizer maintained size, surface charge, coating retention, and in vitro therapeutic efficacy, while slightly reducing early drug release during aerosolization [39]. These findings emphasize that aerosolization is not just a device issue but also a challenge in co-designing the formulation, requiring simultaneous optimization of aerosol performance and physicochemical property stability.

Accordingly, strategies such as spray drying, lyophilization, and the incorporation of protective excipients or polymeric matrices have become essential for developing stable inhalable nanoformulations. For example, it has been reported that spray drying with stabilizers such as trehalose produced powders with moisture contents below 5% while retaining more than 85% of protein activity following aerosolization. However, these outcomes are formulation-, process-, and protein-dependent and should not be considered universally applicable [40]. Similarly, encapsulation within protective polymer matrices like PLGA or modified polyethylene glycol (PEG), including PEGylated lipids, has been shown to reduce particle aggregation and maintain bioactivity for over 24 h in simulated lung fluid. At the same time, the study demonstrated that optimized dry powder formulations exhibited less than 10% dose variability across inhalation flow rates of 30-60 L/min, indicating improved dose consistency under the experimental conditions evaluated. However, this performance is formulation- and device-dependent and should not be generalized to all dry powder inhaler systems [41].

After confirming aerosol formation and formulation stability, the focus shifts to deposition behavior, as the aerodynamic properties of inhaled particles mainly influence their regional distribution within the lung. It is essential to distinguish between primary nanoparticle size and aerodynamic particle size, as these represent distinct yet interconnected design considerations in pulmonary nanomedicine. Primary nanoparticle size affects cargo loading, cellular uptake, intracellular trafficking, mucus interaction, and post-deposition clearance [42]. In contrast, aerodynamic particle size dictates aerosol transport and regional deposition within the respiratory tract [42]. Consequently, nanoscale carriers are frequently integrated into nano-in-micro systems or respirable micron-sized aggregates to attain an MMAD within the optimal range for efficient lung deposition, while facilitating the release or redispersion of the nanoparticle payload following deposition [43,44]. In this context, parameters such as Mass Median Aerodynamic Diameter (MMAD) and Geometric Standard Deviation (GSD), fine-particle fraction, and respirable fraction should be evaluated alongside nanoparticle characteristics when designing inhaled nanomedicines [43,44].

### 3.2. Particle Size-Dependent Lung Deposition

Aerodynamic particle size is a key determinant of pulmonary deposition and is typically characterized by parameters such as MMAD and GSD [45]. Generally, particles with an MMAD of about 1–5 µm are most effective for deep lung delivery. In contrast, larger particles tend to deposit in the oropharyngeal and upper airway regions, and submicron aerosols might be exhaled before reaching the lungs [46]. More specifically, particles larger than 5 µm tend to deposit in the oropharynx and central airways, reducing delivery to the lower respiratory tract. In contrast, particles smaller than 1 µm may exhibit reduced pulmonary deposition because a substantial fraction can be exhaled before deposition. The extent of these exhalation losses depends on factors such as breathing pattern, airway geometry, and particle characteristics, and therefore varies across studies [47]. Regarding GSD, achieving a relatively narrow size distribution below 2.0 is associated with more consistent lung targeting and less variability in pharmacokinetics between patients [48].

Because therapeutic nanoparticles are inherently smaller than 1 µm, they usually do not exhibit optimal aerodynamic behavior for lung deposition on their own. Their delivery therefore often requires reformulation into nano-in-micro systems, spray-dried carrier-based structures, or controlled aggregates with respirable aerodynamic diameters, typically in the 1 to 5 µm range, which subsequently disaggregate or release the nanoscale payload after deposition [48,49]. A representative example is spray-dried chitosan-based inhalable microparticles containing isoniazid, in which a 50–190 kDa chitosan carrier is crosslinked with tripolyphosphate to improve drug retention. These particles are designed to fall within the respirable aerodynamic range of approximately 1–5 µm to enhance deposition in the bronchioles and alveoli, while minimizing proximal deposition of larger particles (>5 µm) and reducing exhalation losses often associated with aerosols smaller than 0.5 µm [42].

The therapeutic goal of inhalation closely relates to the lung region where deposition occurs. Particles with larger MMAD tend to deposit in the upper and central airways, making them more suitable for airway diseases like asthma or bronchitis. Conversely, smaller aerosols are needed for deeper penetration into the small airways, which is important for diseases such as pneumonia [50,51]. Inhalation patterns also influence deposition. Gamma scintigraphy studies have demonstrated that slow inhalation at about 30 L/min improves distal lung delivery compared to faster flow rates [52]. In diseases like pulmonary tuberculosis or metastatic lung cancer, precise alveolar targeting is especially crucial, and ligand-functionalized nanocarriers offer a way to boost uptake by specific pulmonary cell populations [53]. For example, mannosamine-functionalized PLGA-PEG nanoparticles increased macrophage uptake by about 4-fold at 24 h. They improved the antimycobacterial activity of rifapentine, as evidenced by a lower minimum inhibitory concentration than that of the free drug. They also achieved greater intracellular inhibition in infected macrophages, supporting receptor-mediated targeting as an effective way to improve intracellular delivery while reducing nonspecific exposure [53].

However, regional deposition is only one component of successful pulmonary delivery, as subsequent interactions between particles and mucus, surfactant, and resident cells depend strongly on their physicochemical properties.

### 3.3. Physicochemical Properties for Deep Lung Deposition

Beyond aerodynamic size, physicochemical properties significantly affect the success of deep lung delivery [54]. Particles with low density and favorable morphology, such as aerodynamic or near-spherical shapes, typically show better deposition in the alveolar region, while surface charge and hygroscopicity affect mucus penetration, colloidal stability, and post-deposition behavior [54,55]. Surface modification can also influence biological outcomes. For example, in one preclinical study, PEGylation reduced macrophage uptake by more than 60% and prolonged lung retention for up to 24 h. However, these effects are formulation- and model-dependent and may not be generalizable to all applications [56]. Surfactant-mimetic coatings like dipalmitoylphosphatidylcholine (DPPC) can further improve compatibility with the alveolar environment and enhance intrapulmonary distribution; notably, this phospholipid plays a key role in maintaining the extremely low surface tension of the alveolar interface, often below 1 mN/m, throughout the respiratory cycle [57]. Nanocarriers must also resist aggregation during aerosolization and transit, a challenge that can be addressed using hydrophilic stabilizers such as PEG or polysorbate 80 [58]. Meanwhile, stimuli-responsive systems have emerged as a promising strategy to combine effective pulmonary deposition with site-specific drug release. For example, one study reported that a pH-sensitive polymeric nanoparticle formulation released more than 70% of its payload at pH 5.0, compared with less than 20% at pH 7.4, demonstrating preferential drug release under acidic conditions. However, these release profiles are highly dependent on the polymer composition, drug properties, and experimental conditions, and should not be generalized to all pH-responsive nanocarrier systems [59]. Encapsulation of hydrophilic and labile peptide therapeutics into PLGA nanocarriers can also reduce peptide instability and enzymatic degradation, enhance biodistribution and bioavailability, and enable controlled localized delivery. Reported examples include the sustained release of insulin, exenatide, and octreotide, with preservation of peptide activity over extended periods under optimized formulation conditions that involve polymer end groups, stabilizers, and pH control [60].

Consequently, optimizing these physicochemical characteristics is not sufficient; it must be integrated with inhaler device design to ensure efficient aerosol generation, stability, and deep-lung deposition.

### 3.4. Types of Inhalers

The successful translation of optimized physicochemical properties into effective pulmonary delivery depends critically on the choice of inhalation device and its aerosolization mechanism. One of the most widely used inhalation devices is the metered-dose inhaler (MDI), which delivers bronchodilators and corticosteroids because of its portability and quick activation [61]. These propellant-driven devices usually use hydrofluoroalkane (HFA) propellants, and their effectiveness for nanoparticle delivery depends on maintaining stable suspensions without aggregation or nozzle clogging [61]. Recent research has shown HFA-compatible pressurized MDI (pMDI) formulations in which nanoparticles are stabilized using ethanol as a cosolvent and surfactants such as oleic acid to enhance dispersion and aerosol performance; for example, polymeric nanoparticles dispersed in HFA 227 with ethanol achieved a fine particle fraction of 63.5% and an MMAD of 1.6 µm [61]. However, because pressurized MDIs require hand-breath coordination, technique-related errors are still common and can hinder drug delivery. Although spacers can improve administration, error rates remain significant, especially among pediatric and elderly populations, emphasizing the need for ongoing training and, in some cases, alternative inhalation methods [62].

Another widely used class of inhalation devices is the dry powder inhaler (DPI), which differs from MDIs in its propellant-free design and reliance on patient inspiratory effort for drug delivery [63]. For nanoparticle-based systems, co-spray drying with excipients or embedding nanoparticles into inhalable microparticles within the 1 to 5 µm aerodynamic range enables efficient pulmonary deposition, followed by local release of the nanoscale payload [64]. DPIs are propellant-free, portable, and often offer improved chemical stability because the drug remains in the solid state, which can help reduce reliance on cold chain storage. However, their performance is very sensitive to powder engineering and deaggregation behavior. This has led to the development of porous-particle strategies, including phospholipid-based small-pore PulmoSphere™ particles and other engineered carriers, such as nanoporous mannitol, to reduce interparticle cohesion and enhance lung delivery [65]. Similarly, spray-dried formulations that include dispersibility enhancers such as mannitol and surface-active L-leucine can enhance powder de-aggregation and aerosol performance by promoting surface enrichment and reducing moisture-induced clumping, thereby reducing oropharyngeal losses and improving deposition in the lower airways [65].

In contrast to handheld inhalers, nebulization represents an alternative delivery approach that generates aerosols from liquid formulations over an extended period. Nebulized formulations that produce liquid aerosols with jet, ultrasonic, or vibrating mesh devices are especially useful across age groups, particularly in elderly, frail, and pediatric patients, because they require minimal coordination and can support continuous or high-dose delivery [66]. However, for nanoparticle and biologic formulations, nebulization presents significant challenges in preserving physicochemical stability and cargo integrity during aerosol generation [66]. Despite these limitations, nebulized inhalation has proven highly effective at delivering very high local antibiotic concentrations in the lungs while minimizing systemic exposure and toxicity. Approved examples include tobramycin inhalation solution and amikacin liposome inhalation suspension for chronic lung infections [66,67]. Meanwhile, liposomes, polymeric nanoparticles, and other nanocarriers are actively studied in nebulized systems to improve lung deposition, retention, solubility, and cell-specific targeting [66,67].

As a newer generation of inhalation devices, soft mist inhalers (SMI) offer a distinct aerosolization mechanism that enhances deposition efficiency while reducing dependence on patient inhalation effort. SMIs are propellant-free, mechanically activated devices, such as Respimat, that produce a slow-moving fine aerosol cloud, which helps reduce the effort required to inhale, minimizes coordination issues during actuation, and improves dose consistency and lower-airway deposition across different patient groups [68]. Compared with many conventional devices, soft mist inhalers can achieve pulmonary deposition of about 50% while operating largely independently of inspiratory effort, which is especially beneficial for patients unable to produce the minimum inspiratory flow required for DPIs [68,69]. Additionally, the study stated that the Respimat soft mist inhaler generated a fine-particle fraction of approximately 75%, with aerosol particles predominantly ranging from 1 to 5.8 µm, a relatively low cloud velocity of approximately 0.8 m/s at 10 cm from the nozzle, and a spray duration of around 1.5 s. These aerosol characteristics have been associated with reduced oropharyngeal deposition and improved lung deposition under the investigated conditions. However, aerosol performance and deposition may vary depending on the drug formulation, inhalation technique, and patient-specific factors [68,69].

Given the distinct dispersion mechanisms of MDIs, DPIs, nebulizers, and SMI, successful pulmonary delivery depends on matching formulation properties with device-specific performance requirements, since device design directly influences aerosol generation, dose reproducibility, and patient use.

### 3.5. Compatibility and Stability During Aerosolization

In this context, the emitted dose represents the amount of formulation that leaves the device. In contrast, the fine-particle and respirable fractions indicate the proportion of the aerosol likely to reach the lower respiratory tract. Successful integration of nanocarriers into inhalation dosage forms requires closely matching formulation stability during aerosolization with device-specific dispersion behavior. This ensures the delivered aerosol has a respirable MMAD of about 1 to 5 µm and an adequate fine-particle fraction for peripheral lung delivery, while minimizing mouth and throat deposition [70]. In dry powder systems, nanoparticles are often engineered into respirable micron-sized aggregates, such as nanoaggregates or porous nanoparticle aggregates that disperse efficiently during inhalation and then dissociate into primary nanoparticles after deposition [70]. In liquid aerosol systems, colloidal stability is the dominant challenge because nanosuspensions can aggregate without proper stabilizers and must maintain particle size and dispersion during nebulization [70]. Controlled agglomeration into micrometer-sized composite structures is a key particle-engineering strategy because it combines the functional advantages of nanoparticles with the aerodynamic properties required for effective pulmonary delivery [70,71]. Therefore, the performance of any inhaled nanomedicine is ultimately determined not just by the formulation or device alone, but by how well they work together during aerosolization and delivery.

A summary of the major pulmonary dosage forms and their corresponding delivery devices is presented in Table 1.

## 4. Therapeutic Modalities and Nanoparticle Platforms for Pulmonary Drug Delivery

### 4.1. Therapeutic Modalities for Pulmonary Drug Delivery

Small molecules remain the foundation of pulmonary pharmacotherapy because of their well-characterized pharmacokinetics, chemical stability, and comparatively straightforward formulation pathways. Inhaled small-molecule drugs are widely used to treat asthma, COPD, pulmonary infections, and pulmonary hypertension, with bronchodilators and corticosteroids as established examples in clinical practice [72]. As mentioned earlier, pulmonary administration enables rapid pharmacological onset by delivering the drug directly to the site of pathology while reducing systemic exposure and associated adverse effects. This therapeutic efficiency is supported by the large alveolar surface area, thin epithelial barrier, and dense capillary network, which together facilitate rapid absorption into both pulmonary tissue and the systemic circulation [73]. Despite these advantages, the performance of inhaled small molecules may still be limited by mucociliary clearance, enzymatic degradation, and heterogeneous deposition governed by aerodynamic particle size and inhaler characteristics [74]. Nanoparticle-based formulations have therefore emerged as a useful strategy to improve the apparent solubility of poorly water-soluble drugs, prolong lung residence time, and enable controlled or sustained release [75]. Such nanoformulations may also attenuate local irritation and improve the therapeutic index by facilitating targeted delivery to specific pulmonary cell populations, including alveolar macrophages and epithelial cells [76]. Table 2 summarizes different nanoparticle systems and therapeutic modalities used for pulmonary delivery.

Proteins and peptides constitute a rapidly expanding class of therapeutics for pulmonary diseases, encompassing enzymes, monoclonal antibodies, cytokines, and hormone analogs. Their high specificity and potency render them particularly attractive for localized treatment of inflammatory, infectious, and genetic lung disorders [77]. Pulmonary delivery avoids gastrointestinal degradation and hepatic first-pass metabolism, which is especially advantageous for structurally labile biologics. The alveolar epithelium provides a relatively permeable interface that, under appropriate conditions, can facilitate systemic absorption, as demonstrated by inhaled insulin formulations [78]. However, proteins and peptides remain highly susceptible to denaturation, aggregation, and loss of activity during aerosolization and storage, and they are also vulnerable to enzymatic degradation within the lung microenvironment [79]. Nanocarrier systems such as liposomes and polymeric nanoparticles can mitigate these liabilities by protecting cargo structure, stabilizing conformation, and enabling sustained or controlled release. Moreover, surface functionalization with targeting ligands can enhance cellular uptake and receptor-mediated transport, thereby improving therapeutic efficacy and potentially reducing dosing frequency and systemic toxicity [80].

Nucleic acid-based therapeutics, including small interfering RNA (siRNA), messenger RNA (mRNA), and plasmid DNA (pDNA), offer substantial potential for treating pulmonary diseases at the molecular level. These modalities enable gene silencing, protein replacement, or modulation of gene expression directly within lung tissues, providing a mechanistic framework for precision therapy in conditions such as CF, pulmonary fibrosis, and severe asthma [81]. For example, siRNA can selectively downregulate pathogenic genes, whereas mRNA therapeutics can transiently express therapeutic proteins in situ [81]. Unmodified nucleic acids, however, are highly susceptible to nuclease degradation, exhibit poor membrane permeability, and can provoke innate immune activation [82]. Effective pulmonary delivery, therefore, relies on nanoparticle carriers that condense and shield nucleic acids, promote endosomal escape, and mediate efficient cytoplasmic or nuclear delivery. Lipid nanoparticles (LNPs) have demonstrated robust performance in delivering mRNA to the lungs. At the same time, cationic and ionizable polymer systems, such as polyethyleneimine (PEI) derivatives, are being actively explored for gene transfer applications [83]. Critical design parameters include optimizing the aerodynamic particle size (typically 1 to 5 µm for inhaled aerosols) to enable deep lung deposition, minimizing proinflammatory responses, and achieving reproducible gene expression profiles with acceptable safety margins [64].

Pulmonary vaccination has emerged as a promising strategy to induce both mucosal and systemic immunity against respiratory pathogens. The respiratory tract is enriched in antigen-presenting cells, including dendritic cells and alveolar macrophages, which provide an advantageous milieu for priming immune responses against agents such as influenza viruses, coronaviruses, and *Mycobacterium tuberculosis* [84]. Inhaled vaccines can elicit robust mucosal IgA responses in parallel with systemic IgG production, thereby enhancing protection at the primary site of pathogen entry [85]. Nanoparticle platforms enhance vaccine performance by improving antigen stability, enhancing immune cell uptake, and enabling co-delivery of adjuvants to amplify innate and adaptive responses [86]. Dry powder formulations of nanoparticle-based vaccines further improve thermostability and support needle-free administration, which is advantageous for large-scale immunization programs, particularly in resource-limited settings [87]. Remaining challenges involve achieving uniform antigen deposition across the conducting and respiratory zones, limiting excessive local inflammation, and establishing scalable manufacturing processes that preserve antigen structure and immunogenicity throughout production and storage [88].

Gene-editing technologies, particularly Clustered Regularly Interspaced Short Palindromic Repeats (CRISPR)-Cas-based platforms, represent a transformative therapeutic paradigm for inherited and acquired pulmonary diseases. By enabling precise modification of disease-causing alleles, CRISPR-based interventions have the potential to yield durable and, in some cases, curative outcomes for disorders such as CF and alpha-1 antitrypsin deficiency (A1AT) [89]. Pulmonary delivery of gene-editing components, including Cas9 mRNA or protein in combination with guide RNA (gRNA), requires sophisticated nanocarrier systems capable of transient, cell-specific, and safe delivery [90]. Delivery strategies commonly employ LNPs, polymeric nanoparticles, or hybrid nanocarriers engineered to optimize lung deposition, endosomal escape, and nuclear access [91]. Key translational challenges include reducing off-target editing events, tightly controlling editing efficiency, mitigating immunogenicity associated with Cas proteins, and ensuring transient rather than persistent expression to minimize long-term safety risks. Continued advances in nanoparticle design and aerosol engineering are accelerating preclinical development of pulmonary gene-editing therapeutics and are expected to facilitate progression into early-phase clinical studies [92].

**Table 2:** Comparison of therapeutic modalities for nanoparticle systems for pulmonary delivery.

Nanocarrier	Composition	Drug Type	Key Advantages	Limitations	Pulmonary Applications	Reference
Small Molecules	N/A (can be a free drug or encapsulated)	Small-molecule drugs	Rapid action; localized delivery; reduced systemic side effects; improved solubility when nanoformulated	Mucociliary clearance; enzymatic degradation; variable deposition	Asthma; COPD; pulmonary infections; pulmonary hypertension	[76]
Proteins and Peptides	N/A or encapsulated in liposomes/polymeric carriers	Biologics; enzymes; antibodies; cytokines	High specificity; bypasses gastrointestinal degradation; potential systemic absorption	Denaturation; aggregation; enzymatic degradation	Inflammatory lung diseases; enzyme replacement therapy; localized biologic delivery	[80,93,94,95,96,97]
Nucleic Acids	Lipid nanoparticles; polymeric nanoparticles	siRNA; mRNA; plasmid DNA	Protects nucleic acids; enhances cellular uptake; enables gene modulation	Nuclease degradation; immune activation; poor uptake without carrier	Gene silencing; protein replacement; vaccination; genetic lung disorders	[83]
Vaccines	Liposomes; polymeric nanoparticles; dry powder carriers	Antigens; adjuvants	Stimulates mucosal and systemic immunity; enhanced uptake by immune cells; improved stability	Uniform deposition; local inflammation; manufacturing complexity	Pulmonary immunization against influenza, COVID-19, and tuberculosis (TB)	[87]
Gene-Editing Tools	Lipid or polymer-based nanoparticles; hybrid carriers	CRISPR components (Cas9 mRNA/protein; gRNA)	Precise gene modification; targeted delivery; potential curative therapy	Off-target effects; immunogenicity; delivery efficiency	Inherited lung diseases (CF; A1AT deficiency); experimental pulmonary gene therapy	[91]

### 4.2. Nanoparticle Platforms for Pulmonary Drug Delivery

**Figure 2:**
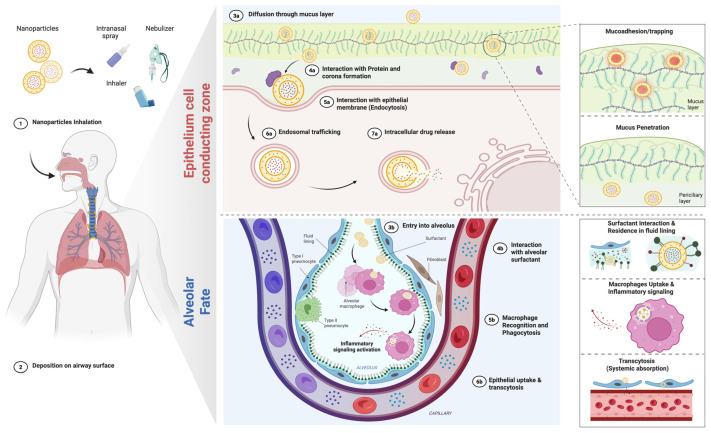
Schematic illustration of nanoparticles’ behavior in the lung following pulmonary administration. (1) Nanoparticles are delivered by nebulizer, inhaler, or intranasal spray and (2) deposit on the airway surface. In the conducting zone, nanoparticles (3a) diffuse through the mucus layer, (4a) interact with proteins and (5a) subsequently undergo endocytic uptake through the epithelial membrane, followed by (6a) endosomal trafficking and (7a) intracellular drug release. In the alveolar region, nanoparticles (3b) enter the alveolar space and may undergo one of three major fates: (4b) inter-act with alveolar surfactant and reside within the alveolar lining fluid and surfactant layer, (5b) recognition and phagocytic uptake by resident alveolar macrophages, leading to phagolysosomal degradation and activation of inflammatory signaling pathways, or (6b) uptake by alveolar epithelial cells and transcytosis across the air–blood barrier, enabling systemic absorption into the bloodstream. Created in BioRender. AlFayez, N. (2026) https://BioRender.com/m2h7azj (accessed on 10 July 2026).

Despite major differences in cargo structure and biological function, shared post-deposition processes that determine nanoparticle transport, cellular uptake, intracellular trafficking, and clearance within the lung often govern the pulmonary performance of nanomedicines. Following inhalation, nanoparticles may diffuse through airway mucus, interact with epithelial membranes, undergo endocytic uptake, or be removed through macrophage-mediated phagocytosis in the alveolar region, with direct consequences for drug release, therapeutic efficacy, and inflammatory signaling (Figure 2).

Liposomes are among the most extensively investigated nanocarriers for pulmonary drug delivery, owing to their biocompatibility, structural resemblance to pulmonary surfactant, and ability to encapsulate both hydrophilic and hydrophobic agents. These phospholipid vesicles can protect sensitive drugs from degradation, improve apparent solubility, and provide sustained release within the lung environment [98]. Surface modification with PEG can prolong residence time in the airways and alveoli, whereas conjugation of targeting ligands can enhance uptake by specific cell populations [99]. Liposomal formulations have demonstrated clinical utility for inhaled antibiotics and anticancer agents, enabling high local drug concentrations while limiting systemic exposure and toxicity [100]. A representative example is the liposomal amikacin formulation (Arikayce^®^), which has been approved for the treatment of refractory *Mycobacterium avium* complex lung infections, demonstrating enhanced lung deposition, prolonged drug retention, and reduced systemic toxicity compared to free amikacin [101]. Nevertheless, key challenges include maintaining structural integrity during nebulization, preventing premature leakage of encapsulated cargo, and scaling up manufacturing in a cost-effective and reproducible manner. Recent advances in lipid composition optimization, manufacturing processes, and aerosolization technologies are improving the stability and clinical feasibility of liposome-based pulmonary therapies [102].

Polymeric nanoparticles offer highly versatile platforms for controlled and targeted pulmonary drug delivery. Constructed from biodegradable polymers such as PLGA or chitosan, these carriers can be engineered to provide tunable release kinetics, high drug loading, and robust structural stability during aerosolization and storage [103]. Stimuli-responsive designs, including pH- or enzyme-sensitive systems, allow site-specific release in response to local pathophysiological cues. Surface modification can enhance mucoadhesion, modulate interaction with mucus and surfactant layers, or facilitate deeper penetration into distal lung regions [104]. In pulmonary applications, polymeric nanoparticles are particularly attractive for the delivery of nucleic acids and protein therapeutics, as they offer protection from degradation and can be tailored to improve cellular internalization and intracellular trafficking [105]. PLGA-based nanoparticles encapsulating siRNA have demonstrated efficient gene silencing in lung tissues, enhanced cellular uptake, and protection of the nucleic acid cargo from enzymatic degradation following pulmonary administration [106]. Polymer selection must balance biocompatibility, degradation kinetics, and the risk of inflammation or chronic lung toxicity. As with other inhaled nanocarriers, careful optimization of particle size, density, and aerodynamic properties is essential to ensure efficient deep-lung deposition and uniform distribution [107].

Solid lipid nanoparticles (SLNs) are composed of a solid lipid core stabilized by surfactants, providing a favorable compromise between biocompatibility and physical stability. SLNs are particularly suitable for encapsulating lipophilic drugs and can provide controlled release through gradual erosion or restructuring of the lipid matrix [108]. Compared with liposomes, SLNs typically exhibit improved physical stability and reduced propensity for drug leakage during storage and nebulization [109]. In the context of pulmonary delivery, SLNs can enhance the apparent solubility of poorly water-soluble compounds, prolong their residence time in the lungs, and protect sensitive cargo during aerosolization [110]. SLN-based formulations of curcumin for pulmonary delivery have demonstrated enhanced solubility, improved lung retention, and increased anti-inflammatory and anticancer activity compared to free curcumin [111]. However, limitations include relatively modest drug-loading capacities and the possibility of polymorphic transitions within the lipid core, which may alter release characteristics and long-term stability. Ongoing formulation efforts focus on optimizing lipid composition and process parameters to overcome these constraints and expand the clinical applicability of SLNs for respiratory therapy [112].

Nanostructured lipid carriers (NLCs) represent a second-generation lipid-based system that addresses several limitations of SLNs by incorporating both solid and liquid lipids into a partially disordered matrix. This structural heterogeneity increases drug accommodation space, enhances drug loading capacity, and reduces the risk of drug expulsion during storage. For pulmonary delivery, NLCs offer improved physical stability, enhanced bioavailability for hydrophobic drugs, and a favorable safety profile [113]. NLCs can be readily functionalized at the surface to support targeted delivery to specific lung cell types or to modulate interactions with mucus and surfactant. Additionally, NLCs can be formulated as nebulizable suspensions or engineered into DPI systems with mass median aerodynamic diameters suitable for deep lung deposition [114]. NLCs loaded with paclitaxel for pulmonary delivery have exhibited enhanced drug solubility, improved deep-lung deposition, prolonged retention, and increased anticancer efficacy in preclinical models compared to conventional formulations [115]. Despite these advantages, rigorous optimization of lipid composition, particle size distribution, and aerosol performance is required to ensure batch-to-batch consistency and predictable therapeutic outcomes [113,116].

Hybrid and emerging nanocarrier platforms integrate the advantages of multiple material classes to address the multifaceted barriers associated with pulmonary drug delivery. Representative systems include lipid-polymer hybrid nanoparticles, inorganic-organic composites, exosome-mimetic vesicles, and sophisticated stimuli-responsive architectures [117]. Lipid-polymer hybrids, for example, combine a polymeric core with a lipid shell to provide enhanced structural stability, controlled drug release, and improved biocompatibility, and have been investigated extensively for lung cancer chemotherapy and gene delivery [118]. These hybrid systems can be engineered to optimize mucus penetration, achieve precise control over drug release profiles, and enable selective uptake by targeted cell populations, making them particularly attractive for complex therapeutics such as nucleic acids, gene-editing tools, and combination regimens [119]. Advanced surface engineering strategies, including cell membrane cloaking and ligand-based targeting, further promote immune evasion and enhance accumulation at diseased sites. Although many of these platforms remain at the preclinical stage, they hold significant potential to overcome current limitations in pulmonary drug delivery by enabling efficient deep lung deposition, sustained therapeutic effects, and minimal off-target toxicity [120]. A representative example is hybrid nanoparticles co-encapsulating doxorubicin and paclitaxel for inhalation therapy, which achieved improved drug stability, controlled release, and enhanced antitumor efficacy in preclinical lung cancer models [121]. Table 3 summarizes the different types of nanoparticle systems intended for pulmonary delivery.

In comparative evaluation, liposomes have advantages as inhaled delivery vehicles because their vesicular structure enables the encapsulation of both hydrophilic therapeutic agents within the aqueous core and hydrophobic agents within the lipid bilayer. Liposomes also have a biocompatible composition, typically based on phospholipids and cholesterol, and have a long-standing clinical precedent among lipid-based nanocarriers [122]. In addition, liposomes can be modified through drug loading, surface targeting, or PEGylation, supporting their adaptability for pulmonary drug delivery applications. This will enhance mucus penetration or alveolar macrophage uptake by engineering the liposome surface, such as through PEGylation [122]. Therefore, many clinical trials and FDA-approved drugs were successfully delivered to the pulmonary system, as summarized in Table 4. The main limitation of liposomes for inhaled delivery is their potential physical instability during aerosolization, particularly during nebulization, where disruptive forces may affect vesicle integrity [109]. 

PLGA and chitosan are promising polymeric nanoparticles for pulmonary delivery due to their ability to protect and transport drugs such as mRNA by providing greater stability against aggregation and degradation. Also, formulation design can be modified by adjusting polymer composition, surface chemistry, and controlled-release properties. [123]. For chitosan, it can interact with anionic mucins due to its positive charge, which in turn increases mucosal interaction time and potentially improves drug absorption [123]. In addition, chitosan can disrupt epithelial tight junctions, which allow greater nanoparticle penetration [123]. This makes chitosan particularly relevant for mucosal vaccines and mucus-rich lung diseases such as cystic fibrosis. Despite the promise of PLGA and chitosan for pulmonary delivery, PLGA/chitosan and other polymeric nanoparticles remain largely preclinical and face several translational limitations. This is due to major biological barriers, such as alveolar macrophages and pulmonary surfactant, which inhibit pulmonary delivery of PLGA and chitosan. Mucus can limit nanoparticle diffusion and promote mucociliary clearance, while alveolar macrophages can rapidly phagocytose deposited particles before they reach target epithelial cells [123]. Pulmonary surfactant may also interact with nanoparticles, promote aggregation, and increase macrophage-mediated clearance [123].

SLNs have advantages as pulmonary nanocarriers because of their solid lipid matrix, which remains stable at body temperature and can encapsulate both hydrophilic and hydrophobic therapeutic agents [74,109]. Compared with liposomes, SLNs offer improved physical stability before and after nebulization, controlled drug release influenced by pH, and greater suitability for industrial scale-up [74,109]. NLCs, as second-generation lipid nanoparticles, were developed to overcome certain SLN limitations by incorporating a less-ordered lipid matrix that improves drug loading, encapsulation efficiency, and release modulation. For pulmonary delivery, both SLNs and NLCs possess nanoscale dimensions that support lower respiratory tract deposition, while their lipophilic and bioadhesive properties may prolong lung residence time and reduce rapid clearance [109]. SLNs and NLCs remain limited by potential drug-loading constraints, lipid-matrix instability or drug expulsion during storage, and the need for further optimization of aerosol performance, sterilization, scalable manufacturing, and repeated-dose pulmonary safety [109].

**Table 4:** Clinical trial- and FDA-approved nanoformulation drugs for pulmonary therapy.

Disease	Carrier	Drug	Commercial Name	Development Stage	Reference
Antibiotic-Resistant Infection	PLGA	Ciprofloxacin	NCT05442736	Early phase I	[124]
Cystic fibrosis (CF)	LNP	cystic fibrosis transmembrane receptor mRNA	MRT5005	Phase I/II clinical trial	[125]
Non-cystic fibrosis bronchiectasis	Lipid micro-particle	Ciprofloxacin	Ciprofloxacin DPI/ BAYQ3939	Two phase III clinical trials completed	[126]
Chronic lung infections with *Pseudomonas aeruginosa* in non-cystic fibrosis bronchiectasis	Liposome	Ciprofloxacin	Apulmiq (Linhaliq/ Pulmaquin)	Two phase III clinical trials completed	[127]
Mycobacterium avium complex lung disease	Liposome	Amikacin	Arikayce^®^	FDA approved	[128]
Chronic pulmonary *Pseudomonas aeruginosa* infection in CF patients.	LNP	Tobramycin	TOBI^®^ Podhaler^®^	FDA approved	[129]
Respiratory distress syndrome in premature infants	Liposome	surfactant-B and -C	Curosurf^®^	FDA approved	[122,130]

## 5. Artificial Intelligence (AI) and In Silico Modeling

### 5.1. AI-Driven Formulation Optimization

The growing burden of chronic respiratory diseases, particularly asthma and COPD, has intensified the need for inhaled therapies that are both mechanistically precise and developmentally efficient [131]. Because the lungs are continuously exposed to environmental stressors, they are especially vulnerable to disease, and this clinical burden also increases the economic pressure to accelerate the development of safe and effective pulmonary formulations [131]. In this context, AI has emerged as a promising tool to address limitations of conventional formulation development, particularly the reliance on empirical trial-and-error approaches that are resource-intensive, slow, and often unable to capture complex multivariable interactions [132,133,134]. Traditional optimization of inhaled formulations is further complicated by incompatibilities among active ingredients, excipients, and processing conditions, which may lead to degradation, reduced efficacy, or altered bioavailability [135]. By contrast, AI and machine learning (ML) models can integrate large datasets incorporating drug properties such as solubility and stability, excipient characteristics, and desired product attributes, enabling the identification of non-obvious relationships between formulation variables and pulmonary performance outcomes such as aerodynamic particle size, fine particle fraction, aerosol stability, regional deposition, lung retention, and local toxicity [135,136]. Unlike one-factor-at-a-time experimental strategies, these models can evaluate multiple variables simultaneously, thereby supporting more efficient formulation refinement [136]. AI-based workflows can also analyze biological datasets and real patient-derived data to assist in target identification, candidate selection, and the prediction of compound behavior under varying conditions, such as temperature and humidity, thereby helping prioritize formulations with greater pharmacological promise [132,136].

Several ML approaches, particularly artificial neural network (ANN) models, have demonstrated encouraging predictive performance in formulation optimization. ANN-based modeling reduced the number of required formulation experiments by approximately 60% compared with conventional experimental approaches. However, the magnitude of this benefit depends on the dataset, formulation complexity, and model design [135]. More broadly, AI is increasingly being applied across the drug development journey, from discovery and formulation design to classification and quality evaluation, thereby shifting effort away from repetitive experimental screening toward data-driven optimization [136,137]. For instance, artificial neural networks and genetic algorithms have been applied to optimize inhalable formulations by predicting solubility, dissolution, aerodynamic performance, and related aerosol attributes [138]. At the same time, Quantitative Structure–Property Relationship (QSPR) models and In Vitro–In Vivo Correlation (IVIVC) tools support stability assessment and the prediction of lung-specific drug behavior [139,140]. DeepDDI and natural language processing (NLP) approaches may assist in identifying drug–drug interactions and extracting relevant evidence from the respiratory literature. In contrast, DeepChem and graph neural networks (GNNs) are being explored to model drug-target interactions, off-target effects, and formulation-property relationships [141,142]. ADMET Predictor^®^, SwissADME, and ProTox-II further assist in evaluating pharmacokinetic and toxicity-related properties, although their outputs should be interpreted with caution for inhaled systems, as they are not inherently specific to aerosolized delivery, local pulmonary exposure, or nanocarrier-dependent behavior [143].

### 5.2. Integrated CFD-PBPK Modeling for Aerosol Deposition and Drug Distribution in the Respiratory System

For inhaled therapeutics, clinical performance depends not only on drug composition but also on where the aerosol deposits within the respiratory tract, making regional deposition a central determinant of efficacy and safety [131,144]. Deposition in non-target regions such as the mouth and throat may reduce lung delivery and produce undesirable local effects, including irritation and inflammation [145]. Although imaging approaches such as single-photon emission computed tomography (SPECT) and gamma scintigraphy have been used to assess pulmonary deposition, these methods are expensive, depend on radioisotope tracers and specialized expertise, and are limited in spatial resolution, particularly when only two-dimensional information is obtained [145]. These limitations have increased interest in computational fluid dynamics models, which can simulate airflow and aerosol deposition in three dimensions across anatomically complex regions such as the larynx and bronchial tree [146]. Despite its effectiveness, numerous computational fluid dynamics (CFD) investigations predominantly focus on the upper and central respiratory regions. They may not fully capture whole-lung deposition, post-deposition processes, or interindividual physiological variability. Although CFD has historically been employed to simulate airflow and particle deposition within the upper respiratory tract owing to the accessibility of high-resolution anatomical data and comparatively reduced computational complexity, recent developments have broadened its scope to include lower airway and whole-lung simulations. Nevertheless, modeling the distal lung remains computationally intensive and necessitates further validation [131,145,147].

In contrast, PBPK describes the processes of absorption, distribution, metabolism, and excretion (ADME) and relies on multiple physiological parameters. However, they do not accurately reflect how the drug is deposited within different areas of the lung. This has led to the emergence of organ-specific pharmacokinetic models (OI-PBPK) to improve the accuracy of predicting the concentrations and efficacy of inhaled drugs [148,149]. To overcome the shortcomings of both CFD and PBPK models, a hybrid model (CFD-PBPK) has been developed to track the inhaled drug from the lungs to its systemic distribution throughout the body. This provides a more accurate overview of the efficacy and safety of the inhaled drug [150,151].

A recent study has applied CFD-PBPK approaches to examine the influence of inhalation pattern and particle size on the systemic absorption of diethylhexyl phthalate (DEHP) and has demonstrated the ability of these models to predict the distribution and accumulation of both the parent compound and its metabolites in the human body [151]. Despite these advances, such hybrid frameworks remain computationally intensive, require substantial amounts of input data, and are difficult to validate directly in humans due to ethical and experimental constraints. Moreover, many existing studies still rely on single-scenario analyses and, therefore, do not yet fully capture interindividual physiological variability [151].

### 5.3. Digital Twins in Pulmonary Diseases

Digital twins have emerged as an increasingly important approach for modeling complex respiratory diseases such as asthma and COPD. These systems are virtual representations of real patients or physiological systems that are continuously updated via data exchange with their physical counterparts, enabling synchronized simulation, analysis, and prediction of disease behavior through integrated cyber-physical modeling and computation [152,153,154]. In respiratory medicine, digital twins integrate physiological, environmental, and clinical data to enable personalized monitoring and early intervention. Emerging research shows they may improve predictive accuracy over traditional models. In pulmonary drug delivery, these models can predict regional deposition, lung exposure, and dosage by including airway geometry, breathing patterns, disease status, and formulation-device characteristics [154]. Their potential value is especially high in diseases such as asthma and COPD, where dynamic environmental variables, including air quality, strongly influence symptoms and exacerbation risk. In principle, integrating geographic data with wearable air-quality sensors could provide continuous, personalized exposure information and improve risk forecasting. At the same time, these benefits are constrained by practical challenges, including dependence on long-term patient adherence to sensor use, the need for continuous data processing, and the difficulty of integrating heterogeneous data streams into routine clinical workflows [152]. Ethical and regulatory concerns surrounding privacy and persistent data capture also remain substantial. Notably, digital twin applications in respiratory medicine remain limited; among proposed models, only six were related to respiratory symptoms, and only three of these included visual patient representations rather than functioning solely as predictive algorithms [152]. This limited implementation highlights both the promise of digital twins and their current developmental immaturity in pulmonary medicine.

### 5.4. Data-Driven Versus Mechanistic Modeling Approaches

Computational modeling in respiratory medicine generally follows either a data-driven or a mechanistic paradigm, each with distinct strengths and limitations [152,155]. Data-driven models rely on existing datasets to identify patterns and generate predictions without explicitly modeling the underlying physiology. These approaches are attractive for handling large data volumes and can provide rapid predictive outputs, for example, when ML is used to predict asthma exacerbation from historical clinical data [156]. However, their performance depends heavily on the quality, representativeness, and update frequency of the input data, and they often provide limited mechanistic insight into the biological processes underlying disease progression or drug response [152,153]. The mechanistic models, by contrast, are grounded in physical, mathematical, and physiological principles and seek to simulate the function of biological systems directly. In the pulmonary setting, such models may describe airflow, regional deposition, transport, or disease-related physiological changes [155]. Their major strengths lie in interpretability and the ability to simulate new or hypothetical scenarios, even when empirical data are incomplete [152,157]. However, these models are often computationally expensive, may require hours to days to run, and are therefore less practical for real-time or routine clinical decision making [152,157]. In practice, the most informative future frameworks may be hybrid models that combine mechanistic interpretability with data-driven adaptability, particularly for pulmonary drug delivery problems that span aerosol physics, formulation behavior, patient variability, and disease progression. For both approaches, reliable application in pulmonary drug delivery depends on a clear definition of the model applicability domain, transparent uncertainty assessment, and validation against experimentally or clinically relevant endpoints [158].

### 5.5. Validation and Regulatory Acceptance of AI-Based Tools

For AI-based tools to become reliable components of pulmonary drug development or clinical decision support, rigorous validation and regulatory acceptance are essential [159,160]. This is particularly important when such tools are used to inform pulmonary endpoints, including MMAD, fine-particle fraction, aerosol stability, regional deposition, lung retention, local toxicity, or patient-specific dosing [161]. Validation must be multidimensional and should establish not only predictive performance but also generalizability, robustness, fairness, and temporal stability. Internal validation evaluates performance on data not used during training but originating from the same environment, thereby providing an initial measure of generalizability beyond the development set [159,162]. External validation extends this assessment to independent datasets from different institutions or sources and is critical for determining whether the model performs reliably across diverse clinical settings [162,163]. In addition, model credibility depends on explicit characterization of uncertainty and a clearly defined applicability domain, particularly when extrapolating across formulation classes, inhalation devices, or patient populations [164].

Temporal validation further examines whether model performance remains stable over time, whereas subgroup validation is required to detect bias and performance disparities across patient groups [155,159,165]. Forward prospective validation, in which the model is applied to future data, is especially valuable because it more closely approximates real clinical use [155,163]. From a regulatory standpoint, the pathway to acceptance in the United States depends on the device’s risk classification, which may be Class I, II, or III. Most AI systems in medical imaging are regulated as Class II devices and typically require a 510(k) submission demonstrating substantial equivalence to a legally marketed predicate device [166,167]. When no suitable predicate exists, the De Novo pathway may be used, allowing classification as Class I or II based on demonstrated safety and effectiveness, and subsequently establishing a regulatory basis for future comparable products [166,167]. Early engagement through the Q-submission program is also recommended to clarify regulatory expectations, identify an appropriate predicate when applicable, and define acceptable testing strategies [166,167]. For pulmonary drug delivery applications, these considerations are particularly important because AI-based tools may influence formulation design, deposition prediction, dose selection, or patient-specific therapeutic decisions, all of which demand both technical rigor and regulatory credibility.

## 6. Barriers to Clinical Translation of Pulmonary Nanomedicine

### 6.1. Toxicity, Stability, and Dose Optimization

The same physicochemical features that enable nanoparticle-mediated pulmonary delivery also underlie many of its principal translational risks. Small size, high surface area, and tunable surface chemistry can improve regional deposition, lung retention, and site-specific targeting within the respiratory tract. Still, they may also promote instability, unintended pulmonary interactions, and toxicity linked to deposition, persistence, or immune activation [14,38,168]. Among the most influential determinants of pulmonary fate are particle size, surface charge, and surface area [168]. Larger particles tend to deposit in the upper or central airways, thus reducing delivery to distal targets and increasing off-target interactions. In contrast, very small particles may be exhaled before deposition, thereby lowering drug delivery efficiency [168,169].

Surface charge is similarly a critical regulator of colloidal stability, cellular uptake, biodistribution, and immune recognition in the lung [170,171]. Positively charged particles, for example, generally interact more strongly with immune cells than negatively charged particles, which may enhance uptake in some settings but may also increase inflammatory risk and compromise safety [172]. In parallel, high surface area systems are often more prone to aggregation and may display less predictable pulmonary behavior, while poor biodegradability can promote prolonged retention, accumulation, and chronic toxicity [38].

Dose control adds a further layer of complexity because therapeutic performance depends not only on nominal dose but also on the fraction of the administered formulation that remains stable during aerosolization, deposits in the intended lung region, and persists long enough to achieve effective exposure. Consequently, pulmonary nanomedicine development requires careful balancing of deposition efficiency, dose reproducibility, colloidal stability, biodegradability, and local tolerability, rather than optimizing any single formulation feature in isolation [170,171,172].

### 6.2. Quality by Design

These challenges have increased the importance of Quality by Design (QbD) as a framework for developing inhaled nanoparticle systems. In this approach, key physicochemical properties of nanocarriers are considered critical quality attributes because they directly influence the quality target product profile, including therapeutic effectiveness, safety, and stability [173]. In pulmonary formulations, these attributes commonly include aerodynamic diameter, particle size distribution, surface charge, encapsulation stability, redispersibility, and batch-to-batch reproducibility, all of which influence both aerosol performance and biological behavior after inhalation [173]. In principle, systematic control of these parameters should enable more robust formulation development and more predictable product quality.

In practice, however, implementation remains challenging because the performance of pulmonary nanoparticles depends on complex, often nonlinear interactions among critical material attributes and process parameters [174,175]. Relatively small changes in composition or processing can alter aerodynamic behavior, formulation stability, and interbatch variability, and these effects are frequently amplified during scale-up [14,175]. Even when design spaces are established using tools such as Design of Experiments (DoE), predictive control remains challenging because lung defense mechanisms and clearance pathways introduce biological variability that conventional QbD models do not readily capture [14,176]. So, successful translation of QbD principles to inhaled nanomedicines requires not only rigorous control of manufacturing variables but also a more integrated understanding of how formulation attributes interact with the pulmonary environment after administration. However, manufacturing control alone might not be sufficient to ensure translational success, because even well-characterized inhaled nanomedicines may elicit complex biological and immunological responses after deposition in the lung.

### 6.3. Immunogenicity and Off-Target Risks

Beyond manufacturing and formulation control, immunogenicity and off-target effects that occur after deposition in the lung further complicate the pulmonary delivery of advanced biologic therapeutics. These therapeutics, including proteins, peptides, nucleic acids, vaccines, and gene editing systems, face unique challenges in pulmonary delivery because their effectiveness depends not only on proper deposition but also on preserving biological activity while avoiding immune responses and off-target effects. After inhalation, these cargos must overcome airway mucus, mucociliary clearance, surfactant interactions, alveolar macrophage uptake, and epithelial transport barriers, all of which can reduce the amount of the administered dose that reaches the target site [38,177]. In inflamed or diseased lungs, these limitations may be further amplified by mucus hypersecretion, airway narrowing, altered surfactant composition, and elevated immune activity, thereby increasing variability in delivery efficiency and therapeutic response [14,22,38]. As a result, higher nominal doses may be required to achieve effective local concentrations, which can in turn increase the risk of local toxicity, systemic exposure, and unintended biological effects, particularly in patients with impaired pulmonary function [22,38,177].

These concerns are particularly important for advanced biologic cargoes because pulmonary delivery may provoke both carrier-related and cargo-related immune responses. Nanoparticle composition, size, charge, and persistence can affect immune recognition, inflammatory signaling, and long-term tolerability. Simultaneously, the biologic payload itself may pose additional risks such as immunogenicity, heightened innate immune activation, or off-target functional effects [177]. In heterogeneous diseases such as lung cancer, pulmonary tuberculosis, and chronic inflammatory lung disorders, anatomical distortion, mucus accumulation, and altered breathing patterns can further compromise target engagement and increase nonspecific deposition [177]. Moreover, reports that inhalable nanoparticles such as nano silver, Si_3_N_4_, Fe_2_O_3_, ZrO_2_, Al_2_O_3_, TiO_2_, chrysotile, silica-based nanoparticles, and quantum dots can induce epithelial injury, mitochondrial dysfunction, immune cell impairment, inflammation, oxidative stress, fibrosis, and genotoxic effects in vitro and in vivo underscore the need for careful material selection and rigorous safety evaluation [178,179]. These risks may become even more pronounced in chronic inflammatory lung disease, where altered biomechanics and reduced clearance can increase pulmonary retention and extrapulmonary translocation [178,179]. These considerations highlight that successful pulmonary translation of advanced biologic therapeutics requires not only efficient delivery but also careful control of immunogenicity, off-target exposure, and long-term toxicological risk.

### 6.4. Regulatory Challenges

Despite strong preclinical interest, translating inhaled nanoparticle systems into clinically approved products remains slow due to major scientific, manufacturing, regulatory, and market access barriers [173]. Formulations that perform well at laboratory scale frequently fail during scale-up because particle size, morphology, zeta potential, drug loading, and aerosol behavior may shift as manufacturing processes become more complex and less tightly controlled [180]. Production of pulmonary nanomedicines often involves multistep processes, including nanoparticle formation, homogenization, spray drying, milling, extrusion, and fluidized bed coating. Each step can introduce variability and affect reconstitution behavior, aerosolization efficiency, and batch-to-batch reproducibility [180]. Regulatory agencies such as the U.S. Food and Drug Administration and the European Medicines Agency, therefore, require robust evidence of safety, efficacy, quality and manufacturing consistency. This includes a thorough evaluation of toxicity, immunogenicity, and the risk of nanoparticle accumulation after repeated exposure [14,181]. However, guidance documents specifically tailored to inhaled nanomedicines remain limited, creating uncertainty in development strategy and often necessitating additional studies on ADME [181].

Translation is further complicated by the limited predictive value of many in vitro, ex vivo, and animal models, which do not always reflect human pulmonary responses with sufficient accuracy, and by the observation that many promising formulations fail before or during clinical evaluation for reasons that remain poorly understood [182]. A more detailed understanding of nanoparticle biodistribution, intracellular trafficking, and long-term fate in the human lung will therefore be essential for both regulatory confidence and clinical progress [182]. Device compatibility introduces an additional layer of complexity because therapeutic performance depends not only on the formulation itself but also on its interaction with the selected inhalation platform. Devices can affect particle stability, entrapment efficiency, agglomeration, concentration, viscosity, fine particle fraction, aerosol deposition, and resistance to shear, and some systems, particularly pressurized metered dose inhalers, may be less suitable for certain nanoparticle formulations [170,183].

Practical considerations also shape translation. Some inhalable nanomedicines may prove costly, unstable, difficult to manufacture, or complex to integrate into routine clinical practice, while physician acceptance, patient adherence, reimbursement, and competition with established therapies further influence market viability [184,185]. For dry powder inhalers in particular, formulation and drying strategies must be carefully optimized to preserve nanoparticle integrity and maximize deep lung deposition [184,185]. The clinical translation of pulmonary nanomedicines remains constrained by challenges related to scalable manufacturing, device compatibility, long-term safety evaluation, and regulatory uncertainty. Progress depends on standardized methods, better preclinical models, and clearer regulations to enable approval and wider adoption of inhaled nanoparticle therapies.

## 7. Conclusions

Pulmonary drug delivery is vital for local and systemic therapy because of its non-invasive nature, rapid onset, high local drug levels, and ability to avoid gastrointestinal breakdown and liver metabolism. With the growth of inhaled biologics, nucleic acid therapies, and nanomedicine, a framework addressing biological barriers, formulation, aerosol engineering, modeling, and translation is increasingly necessary. However, complex barriers such as regional deposit variability, mucociliary clearance, macrophage uptake, transport limitations, enzymatic degradation, and disease-related changes hinder the full therapeutic potential by reducing drug retention, bioavailability, and consistency. This review shows how advances in nanomedicine could improve drug stability, mucus penetration, controlled release, and targeted delivery for various therapeutics. Aerosol engineering, including aerodynamic optimization, formulation-device compatibility, and maintaining stability during aerosolization, is also needed for successful clinical use. We also emphasize the expanding role of computational approaches, including AI, ML, CFD, PBPK modeling, and digital twin frameworks, in reducing reliance on empirical formulation development, improving predictions of pulmonary deposition and systemic exposure, and enabling more precise inhaled therapies. Despite these advances, major challenges related to long-term safety, immunogenicity, scalable manufacturing, regulatory uncertainty, and market adoption remain. Future progress depends on merging nanotechnology, aerosol engineering, computational modeling, regulation, and manufacturing to turn safer, precise inhaled therapies into clinical practice for respiratory infections, inflammatory diseases, fibrosis, genetic disorders, and lung cancer. This marks a shift from traditional inhaled medicines to precision pulmonary treatments, using personalized approaches for improved therapy.

## Figures and Tables

**Table 1:** Pulmonary dosage forms and delivery devices.

Dosage Form	Device	Particle Size Range	Advantages	Limitations	Reference
Propellant-driven aerosol, solution, or suspension	MDI, HFA pMDI	Aerosol MMAD is typically 1 to 5 µm; for example, MMAD 1.6 µm with a fine particle fraction of 63.5%	Portable, rapid actuation, clinically established; can achieve respirable aerosols with high fine particle fractions in optimized HFA systems	Requires hand-breath coordination; technique errors reduce delivery; nanoparticle suspensions must avoid aggregation and nozzle blockage	[61,62]
Dry respirable powder, carrier-based or engineered nano in micro composite	DPI	Aerodynamic MMAD is typically 1 to 5 µm; often engineered in the 2 to 4 µm range for post-deposition nanoparticle release	Propellant-free, portable, improved solid-state stability; porous carriers and dispersibility enhancers such as PulmoSphere™, nanoporous mannitol, and L-leucine improve deaggregation and lung delivery	Performance depends on inspiratory flow, powder engineering, and moisture control; user dependence can affect dose reproducibility	[41,48,49,65]
Liquid aerosol, solution, or nanosuspension	Nebulizer, jet, ultrasonic, or vibrating mesh	Droplet or aerosol aerodynamic diameter is typically 1 to 5 µm for deep lung delivery	Minimal coordination required; suitable for pediatric, frail, and elderly patients; supports continuous or high-dose delivery; approved examples include tobramycin inhalation solution and amikacin liposome inhalation suspension	Nanocarrier and biologic formulations must preserve physicochemical stability and drug integrity during aerosolization; shear and interfacial stress may alter size or bioactivity	[39,66,67]
Slow-moving soft mist aerosol	SMI, e.g., Respimat)	Fine particle fraction about 75%; particles greater than 1 µm and less than 5.8 µm; lung deposition up to about 50%	Propellant-free; slow plume improves lower airway delivery and reduces oropharyngeal loss; less dependent on inspiratory effort than DPI systems	Device technique still affects delivery; experience with nanoparticle formulations remains more limited than for MDIs, DPIs, or nebulizers	[68,69]

**Table 3:** Comparison of nanoparticle platforms for pulmonary drug delivery.

Nanocarrier	Composition	Drug Type	Key Advantages	Limitations	Pulmonary Applications	Reference
Liposomes	Phospholipid bilayers	Small molecules; proteins; vaccines	Biocompatible; protects labile drugs; sustained release; surface modification	Instability during nebulization; drug leakage; scale-up challenges	Antibiotic therapy, anticancer agents, protein delivery	[100]
Polymeric Nanoparticles	Biodegradable polymers (PLGA; chitosan)	Small molecules; proteins; nucleic acids	Controlled release; high loading; tunable size; stimulus-responsive	Potential inflammation; formulation complexity	Protein/gene delivery; chronic lung disease therapy	[105]
Solid Lipid Nanoparticles (SLNs)	Solid lipid core + surfactant	Lipophilic drugs	Controlled release; enhanced stability vs. liposomes; protects the drug	Low drug loading; polymorphic transitions	Hydrophobic drug delivery; local pulmonary therapy	[110]
Nanostructured Lipid Carriers (NLCs)	Solid + liquid lipid matrix	Lipophilic drugs	High drug loading; improved release control; stable; scalable	Requires lipid optimization; aerosol performance critical	Pulmonary drug delivery; dry powder inhalation; chronic lung therapy	[114]
Hybrid/Emerging Nanocarriers	Lipid-polymer hybrids; inorganic-organic composites; exosome-mimetic	Nucleic acids; gene-editing tools; combination drugs	Enhanced stability; targeted delivery; mucus penetration; stimuli-responsive release	Mostly preclinical; complex design; regulatory hurdles	Advanced gene therapy, combination therapy, and experimental pulmonary treatments	[119,120]

## Data Availability

No new data were created or analyzed in this study. Data sharing is not applicable to this article.

## References

[1] He S., Gui J., Xiong K., Chen M., Gao H., Fu Y. (2022). A roadmap to pulmonary delivery strategies for the treatment of infectious lung diseases. J. Nanobiotechnol..

[2] Costa M.P., Abdu J.O.C., Moura M.F.C.S., Silva A.C., Zacaron T.M., Paiva M.R.B., Fabri R.L., Pittella F., Perrone Í.T., Tavares G.D. (2025). Exploring the Potential of PLGA Nanoparticles for Enhancing Pulmonary Drug Delivery. Mol. Pharm..

[3] Yong J., Shu H., Zhang X., Yang K., Luo G., Yu L., Li J., Huang H. (2024). Natural Products-Based Inhaled Formulations for Treating Pulmonary Diseases. Int. J. Nanomed..

[4] Petersson G. (2025). Expanding Opportunities for Inhaled Drug Delivery. ONdrugDelivery.

[5] Rahman Sabuj M.Z., Islam N. (2021). Inhaled antibiotic-loaded polymeric nanoparticles for the management of lower respiratory tract infections. Nanoscale Adv..

[6] Fernández-García R., Fraguas-Sánchez A.I. (2024). Nanomedicines for Pulmonary Drug Delivery: Overcoming Barriers in the Treatment of Respiratory Infections and Lung Cancer. Pharmaceutics.

[7] Parvin N., Aslam M., Alam M.N., Mandal T.K. (2025). Nanotechnology Driven Innovations in Modern Pharmaceutics: Therapeutics, Imaging, and Regeneration. Nanomaterials.

[8] Kumar M., Hilles A.R., Almurisi S.H., Bhatia A., Mahmood S. (2023). Micro and nano-carriers-based pulmonary drug delivery system: Their current updates, challenges, and limitations—A review. JCIS Open.

[9] Alshehri A.A., Aodah A.H., Alradwan I.A., Alnefaie M.K., Nassar M.S., Alduhaymi I.S., Aldossary A.M., Al Fayez N., Tawfik E.A., Almughem F.A. (2026). Bacteriophages as Therapeutic Agents for Pulmonary Infections: From Biological Principles to Clinical Applications. Pharmaceutics.

[10] Fan Y., Zhou Y., Zhao J., Zhao Y. (2025). Advances in Inhaled Nanoparticle Drug Delivery for Pulmonary Disease Management. FASEB J. Off. Publ. Fed. Am. Soc. Exp. Biol..

[11] Politakos N., Gregoriou V.G., Chochos C.L. (2024). Pulmonary Drug Delivery through Responsive Materials. Macromol.

[12] Cao J., Xu Y., Zhang J., Fang T., Wu F., Zhen Y., Yu X., Liu Y., Li J., Dongkai W. (2024). “Nano-in-Micro” Structured Dry Powder Inhalers for pulmonary delivery: Advances and challenges. J. Drug Deliv. Sci. Technol..

[13] Chen D., Liu J., Wu J., Suk J.S. (2021). Enhancing nanoparticle penetration through airway mucus to improve dru g delivery efficacy in the lung. Expert Opin. Drug Deliv..

[14] Sakkal M., Abdelmoteleb R.W.A., Al Ali A., Jardan Y.A.B., Löbenberg R., Sarfraz M. (2025). Inhalable nanoparticle-based drug delivery system for non-small cell lung cancer therapy: Promises and challenges. Saudi Pharm. J..

[15] Guo Y., Bera H., Shi C., Zhang L., Cun D., Yang M. (2021). Pharmaceutical strategies to extend pulmonary exposure of inhaled medicines. Acta Pharm. Sin. B.

[16] Islam M.S., Paul G., Ong H.X., Young P.M., Gu Y.T., Saha S.C. (2020). A Review of Respiratory Anatomical Development, Air Flow Characterization and Particle Deposition. Int. J. Environ. Res. Public Health.

[17] Ibrahim M., Garcia-Contreras L. (2013). Mechanisms of absorption and elimination of drugs administered by inhalation. Ther. Deliv..

[18] Massaro G.D., Massaro D. (1996). Formation of pulmonary alveoli and gas-exchange surface area: Quantitation and regulation. Annu. Rev. Physiol..

[19] Han X., Zhang E., Shi Y., Song B., Du H., Cao Z. (2019). Biomaterial-tight junction interaction and potential impacts. J. Mater. Chem. B.

[20] Yue P., Zhou W., Huang G., Lei F., Chen Y., Ma Z., Chen L., Yang M. (2022). Nanocrystals based pulmonary inhalation delivery system: Advance and challenge. Drug Deliv..

[21] Roth D., Şahin A.T., Ling F., Tepho N., Senger C.N., Quiroz E.J., Calvert B.A., van der Does A.M., Güney T.G., Glasl S. (2025). Structure and function relationships of mucociliary clearance in human and rat airways. Nat. Commun..

[22] Liang W., Pan H.W., Vllasaliu D., Lam J.K.W. (2020). Pulmonary Delivery of Biological Drugs. Pharmaceutics.

[23] Malachowski T., Hassel A. (2020). Engineering nanoparticles to overcome immunological barriers for enhanced drug delivery. Eng. Regen..

[24] Enlo-Scott Z., Bäckström E., Mudway I., Forbes B. (2021). Drug metabolism in the lungs: Opportunities for optimising inhaled medicines. Expert Opin. Drug Metab. Toxicol..

[25] Zhao M., Ma J., Li M., Zhang Y., Jiang B., Zhao X., Huai C., Shen L., Zhang N., He L. (2021). Cytochrome P450 Enzymes and Drug Metabolism in Humans. Int. J. Mol. Sci..

[26] Matthews A.A., Ee P.L.R., Ge R. (2020). Developing inhaled protein therapeutics for lung diseases. Mol. Biomed..

[27] Pan M., Zhou X. (2025). Airway remodeling in chronic obstructive pulmonary disease: Characteristics and opportunities. Front. Med..

[28] Savin I.A., Zenkova M.A., Sen’kova A.V. (2023). Bronchial Asthma, Airway Remodeling and Lung Fibrosis as Successive Steps of One Process. Int. J. Mol. Sci..

[29] Darquenne C., Corcoran T.E., Lavorini F., Sorano A., Usmani O.S. (2024). The effects of airway disease on the deposition of inhaled drugs. Expert Opin. Drug Deliv..

[30] Prasher P., Sharma M., Singh S.K., Gulati M., Jha N.K., Gupta P.K., Gupta G., Chellappan D.K., Zacconi F., de Jesus Andreoli Pinto T. (2022). Targeting mucus barrier in respiratory diseases by chemically modified advanced delivery systems. Chem.-Biol. Interact..

[31] Wang H., Xian J., Zhu G., Xie Z., Du G., Zhang Y., Yang L., Zhang Y., Li T., Zhou Y. (2026). Inhalational therapy of pulmonary infection via macrophage-targeted nanoemulsions. Acta Pharm. Sin. B.

[32] Azhdarifam S., Metanat M., Ghobeishavi N., Nouroloyouni S., Esfahlan R.J. (2026). Nanoparticle-based strategies for immune modulation and reorganization in the tumor microenvironment to combat cancer cells. Biomed. Pharmacother..

[33] Shaibie N.A., Mohammad Faizal N.D.F., Buang F., Srichana T., Mohd Amin M.C.I. (2025). Inhaled biologics for respiratory diseases: Clinical potential and emerging technologies. Drug Deliv. Transl. Res..

[34] Rhee C.K., Yoshisue H., Lad R. (2019). Fixed-Dose Combinations of Long-Acting Bronchodilators for the Management of COPD: Global and Asian Perspectives. Adv. Ther..

[35] Qin L., Cui Z., Wu Y., Wang H., Zhang X., Guan J., Mao S. (2023). Challenges and Strategies to Enhance the Systemic Absorption of Inhaled Peptides and Proteins. Pharm. Res..

[36] Sutar Y., Nabeela S., Singh S., Alqarihi A., Solis N., Ghebremariam T., Filler S., Ibrahim A.S., Date A., Uppuluri P. (2022). Niclosamide-loaded nanoparticles disrupt Candida biofilms and protect mice from mucosal candidiasis. PLoS Biol..

[37] Berkenfeld K., Carneiro S., Corzo C., Laffleur F., Salar-Behzadi S., Winkeljann B., Esfahani G. (2024). Formulation strategies, preparation methods, and devices for pulmonary delivery of biologics. Eur. J. Pharm. Biopharm..

[38] Mahmoud D.E., Hosseini S.H., Rathore H.A., Alkilany A.M., Heise A., Elhissi A. (2025). Inhalable Nanotechnology-Based Drug Delivery Systems for the Treatment of Inflammatory Lung Diseases. Pharmaceutics.

[39] Gonsalves A., Menon J.U. (2024). Impact of Nebulization on the Physicochemical Properties of Polymer-Lipid Hybrid Nanoparticles for Pulmonary Drug Delivery. Int. J. Mol. Sci..

[40] Omidian H., Nokhodchi A., Babanejad N. (2025). Dry Powder Inhalers for Delivery of Synthetic Biomolecules. Pharmaceuticals.

[41] Encinas-Basurto D., Martinez-Flores P.D., García J., Lopez-Mata M.A., García-González G., Rodea G.E., Eedara B.B., Mansour H.M., Juarez J. (2025). Latest Advances in Inhalable Dry Powder Bacteriophage Therapy for Pulmonary Infections. Pharmaceutics.

[42] Pasero L., Susa F., Limongi T., Pisano R. (2024). A Review on Micro and Nanoengineering in Powder-Based Pulmonary Drug Delivery. Int. J. Pharm..

[43] Finlay W.H., Darquenne C. (2020). Particle Size Distributions. J. Aerosol Med. Pulm. Drug Deliv..

[44] Vehring R., Foss W.R., Lechuga-Ballesteros D. (2007). Particle formation in spray drying. J. Aerosol Sci..

[45] Muralidharan P., Malapit M., Mallory E., Hayes D., Mansour H.M. (2015). Inhalable nanoparticulate powders for respiratory delivery. Nanomed. Nanotechnol. Biol. Med..

[46] Chaurasiya B., Zhao Y.Y. (2020). Dry Powder for Pulmonary Delivery: A Comprehensive Review. Pharmaceutics.

[47] Cheng D., Pan T., Wang X., Tian R., Fan H., Wei L., He S., Dong R., Yan X., Wu M.X. (2025). An advanced inhalable dry powder, mucus-penetrating aerosol platform: Bridging Andrographolide delivery with clinical translation. Biomaterials.

[48] Kaur R., Kaur R., Singh C., Kaur S., Goyal A.K., Singh K.K., Singh B. (2019). Inhalational drug delivery in pulmonary aspergillosis. Crit. Rev. Ther. Drug Carr. Syst..

[49] Chan H.W., Chow S., Zhang X., Zhao Y., Tong H.H.Y., Chow S.F. (2023). Inhalable Nanoparticle-based Dry Powder Formulations for Respiratory Diseases: Challenges and Strategies for Translational Research. AAPS PharmSciTech.

[50] Hickey A.J., Pena E.S., Maloney Norcross S.E. (2025). Aerosol particle design to promote targeting in the respiratory tract. Eur. J. Pharm. Sci..

[51] Fernández Tena A., Casan Clarà P. (2012). Deposition of inhaled particles in the lungs. Arch. Bronconeumol..

[52] Usmani O.S., Roche N., Jenkins M., Stjepanovic N., Mack P., De Backer W. (2021). Consistent Pulmonary Drug Delivery with Whole Lung Deposition Using the Aerosphere Inhaler: A Review of the Evidence. Int. J. Chronic Obstr. Pulm. Dis..

[53] Luan H., Peng C., Yasin P., Shang Q., Xiang W., Song X. (2025). Mannosamine-Engineered Nanoparticles for Precision Rifapentine Delivery to Macrophages: Advancing Targeted Therapy Against Mycobacterium Tuberculosis. Drug Des. Dev. Ther..

[54] Dhoble S., Kapse A., Ghegade V., Chogale M., Ghodake V., Patravale V., Vora L.K. (2024). Design, development, and technical considerations for dry powder inhaler devices. Drug Discov. Today.

[55] Huang G., Shuai S., Zhou W., Chen Y., Shen B., Yue P. (2022). To Enhance Mucus Penetration and Lung Absorption of Drug by Inhalable Nanocrystals-In-Microparticles. Pharmaceutics.

[56] Shen T.W., Fromen C.A., Kai M.P., Luft J.C., Rahhal T.B., Robbins G.R., DeSimone J.M. (2015). Distribution and Cellular Uptake of PEGylated Polymeric Particles in the Lung Towards Cell-Specific Targeted Delivery. Pharm. Res..

[57] Han S., Mallampalli R.K. (2015). The Role of Surfactant in Lung Disease and Host Defense against Pulmonary Infections. Ann. Am. Thorac. Soc..

[58] Gressler S., Hipfinger C., Part F., Pavlicek A., Zafiu C., Giese B. (2025). A systematic review of nanocarriers used in medicine and beyond—Definition and categorization framework. J. Nanobiotechnol..

[59] Das S.S., Bharadwaj P., Bilal M., Barani M., Rahdar A., Taboada P., Bungau S., Kyzas G.Z. (2020). Stimuli-Responsive Polymeric Nanocarriers for Drug Delivery, Imaging, and Theragnosis. Polymers.

[60] Omidian H., Wilson R.L., Castejon A.M. (2025). Recent Advances in Peptide-Loaded PLGA Nanocarriers for Drug Delivery and Regenerative Medicine. Pharmaceuticals.

[61] Kumar R., Mehta P., Shankar K.R., Rajora M.A.K., Mishra Y.K., Mostafavi E., Kaushik A. (2022). Nanotechnology-Assisted Metered-Dose Inhalers (MDIs) for High-Performance Pulmonary Drug Delivery Applications. Pharm. Res..

[62] Cho-Reyes S., Celli B.R., Dembek C., Yeh K., Navaie M. (2019). Inhalation Technique Errors with Metered-Dose Inhalers Among Patients with Obstructive Lung Diseases: A Systematic Review and Meta-Analysis of U.S. Studies. Chronic Obstr. Pulm. Dis..

[63] Levy M.L., Carroll W., Izquierdo Alonso J.L., Keller C., Lavorini F., Lehtimäki L. (2019). Understanding Dry Powder Inhalers: Key Technical and Patient Preference Attributes. Adv. Ther..

[64] Negi A., Nimbkar S., Moses J.A. (2023). Engineering Inhalable Therapeutic Particles: Conventional and Emerging Approaches. Pharmaceutics.

[65] Shetty N., Cipolla D., Park H., Zhou Q.T. (2020). Physical stability of dry powder inhaler formulations. Expert Opin. Drug Deliv..

[66] Yan R., Zou C., Yang X., Zhuang W., Huang Y., Zheng X., Hu J., Liao L., Yao Y., Sun X. (2025). Nebulized inhalation drug delivery: Clinical applications and advancements in research. J. Mater. Chem. B.

[67] Sempere A., Los-Arcos I., Sacanell J., Berastegui C., Campany-Herrero D., Vima J., Martín-Gómez M.T., Sánchez L., Martínez-González D., Bravo C. (2024). Tobramycin Systemic Absorption in Lung Transplant Recipients Treated With Inhaled Tobramycin: A Cohort Study. Transpl. Int. Off. J. Eur. Soc. Organ Transplant..

[68] Muruganantham S., Ramesh S.L. (2025). Soft mist, dry powder, and smart inhalers: Comparative technologies and clinical impact on asthma and COPD management. Egypt. J. Bronchol..

[69] Anzueto A., Miravitlles M. (2020). Tiotropium in chronic obstructive pulmonary disease—A review of clinical development. Respir. Res..

[70] Mansour H.M., Muralidharan P., Hayes D. (2024). Inhaled Nanoparticulate Systems: Composition, Manufacture and Aerosol Delivery. J. Aerosol Med. Pulm. Drug Deliv..

[71] Malamatari M., Charisi A., Malamataris S., Kachrimanis K., Nikolakakis I. (2020). Spray Drying for the Preparation of Nanoparticle-Based Drug Formulations as Dry Powders for Inhalation. Processes.

[72] Kumar Subramani P.P.N.R., Narayanasamy D. (2024). The Role of Pulmonary Drug Delivery in Modern Therapeutics: An Overview. Cureus.

[73] Wang B., Wang L., Yang Q., Zhang Y., Qinglai T., Yang X., Xiao Z., Lei L., Li S. (2024). Pulmonary inhalation for disease treatment: Basic research and clinical translations. Mater. Today Bio.

[74] El-Sherbiny I.M., El-Baz N.M., Yacoub M.H. (2015). Inhaled nano- and microparticles for drug delivery. Glob. Cardiol. Sci. Pract..

[75] Liu Y., Liang Y., Yuhong J., Xin P., Han J.L., Du Y., Yu X., Zhu R., Zhang M., Chen W. (2024). Advances in Nanotechnology for Enhancing the Solubility and Bioavailability of Poorly Soluble Drugs. Drug Des. Dev. Ther..

[76] Bakrim S., Khalid A., Abdalla A.N., Ibrahim S.E., Hamza S.M.A., El Omari N., Wen G.K., Lee L.-H., Bouyahya A. (2026). Encapsulation-based enhancements in modern drug delivery systems. Int. J. Pharm..

[77] Qi Y., Yang B., Ouyang H., Wang X., Li C., Li L., Zhang J. (2025). Advanced nanotherapies for precision treatment of inflammatory lung diseases. Bioact. Mater..

[78] Nana S., Govender M., Choonara Y.E. (2025). Modified-Release Pulmonary Delivery Systems for Labile Bioactives: Design, Development, and Applications. Pharmaceutics.

[79] Liu M., Svirskis D., Proft T., Loh J., Yin N., Li H., Li D., Zhou Y., Chen S., Song L. (2025). Progress in peptide and protein therapeutics: Challenges and strategies. Acta Pharm. Sin. B.

[80] Su S., Peter M.K. (2020). Recent Advances in Nanocarrier-Assisted Therapeutics Delivery Systems. Pharmaceutics.

[81] Liu M., Wang Y., Zhang Y., Hu D., Tang L., Zhou B., Yang L. (2025). Landscape of small nucleic acid therapeutics: Moving from the bench to the clinic as next-generation medicines. Signal Transduct. Target. Ther..

[82] Santa P., Garreau A., Serpas L., Ferriere A., Blanco P., Soni C., Sisirak V. (2021). The Role of Nucleases and Nucleic Acid Editing Enzymes in the Regulation of Self-Nucleic Acid Sensing. Front. Immunol..

[83] Qin Y., Ou L., Zha L., Zeng Y., Li L. (2023). Delivery of nucleic acids using nanomaterials. Mol. Biomed..

[84] Mettelman R.C., Allen E.K., Thomas P.G. (2022). Mucosal immune responses to infection and vaccination in the respiratory tract. Immunity.

[85] Lin Y., Hu Z., Fu Y.-X., Peng H. (2024). Mucosal vaccine development for respiratory viral infections. hLife.

[86] Ahmadivand S., Gomez-Casado E. (2026). Advances in Nanoparticles as Vaccine Adjuvants. Vaccines.

[87] Loo C.Y., Lee W.H., Zhou Q.T. (2023). Recent Advances in Inhaled Nanoformulations of Vaccines and Therapeutics Targeting Respiratory Viral Infections. Pharm. Res..

[88] Zhuo Y., Zeng H., Su C., Lv Q., Cheng T., Lei L. (2024). Tailoring biomaterials for vaccine delivery. J. Nanobiotechnol..

[89] Abdelnour S.A., Xie L., Hassanin A.A., Zuo E., Lu Y. (2021). The Potential of CRISPR/Cas9 Gene Editing as a Treatment Strategy for Inherited Diseases. Front. Cell Dev. Biol..

[90] Guzman Gonzalez V., Grunenberger A., Nicoud O., Czuba E., Vollaire J., Josserand V., Le Guével X., Desai N., Coll J.-L., Divita G. (2024). Enhanced CRISPR-Cas9 RNA system delivery using cell penetrating peptides-based nanoparticles for efficient in vitro and in vivo applications. J. Control. Release.

[91] Jung H.N., Lee S.Y., Lee S., Youn H., Im H.J. (2022). Lipid nanoparticles for delivery of RNA therapeutics: Current status and the role of in vivo imaging. Theranostics.

[92] Kalter N., Fuster-García C., Silva A., Ronco-Díaz V., Roncelli S., Turchiano G., Gorodkin J., Cathomen T., Benabdellah K., Lee C. (2025). Off-target effects in CRISPR-Cas genome editing for human therapeutics: Progress and challenges. Mol. Ther. Nucleic Acids.

[93] Zhang S., Ahn J. (2025). Phage Therapy as a Novel Alternative to Antibiotics Through Adaptive Evolution and Fitness Trade-Offs. Antibiotics.

[94] Burki T. (2020). A new paradigm for drug development. Lancet Digit. Health.

[95] Kokudeva M., Vichev M., Naseva E., Miteva D.G., Velikova T. (2024). Artificial intelligence as a tool in drug discovery and development. World J. Exp. Med..

[96] Richardson P., Griffin I., Tucker C., Smith D., Oechsle O., Phelan A., Rawling M., Savory E., Stebbing J. (2020). Baricitinib as potential treatment for 2019-nCoV acute respiratory disease. Lancet.

[97] Qian Y., Zhu Z., Zhu J., Chen L., Du H. (2025). Phage therapy: Innovative approaches for refractory pulmonary infections. Virus Res..

[98] Zhang X., Tu S., Tian J., Liang Y., An Y., Zhang T., Guan H., Xiong B., Qin L., Li Y. (2025). Liposome-Based Nanoparticle Delivery Systems for Lung Diseases: Opportunities and Challenges. Int. J. Nanomed..

[99] Suk J.S., Xu Q., Kim N., Hanes J., Ensign L.M. (2016). PEGylation as a strategy for improving nanoparticle-based drug and gene delivery. Adv. Drug Deliv. Rev..

[100] Bassetti M., Vena A., Russo A., Peghin M. (2020). Inhaled Liposomal Antimicrobial Delivery in Lung Infections. Drugs.

[101] Zhang J., Leifer F., Rose S., Chun D.Y., Thaisz J., Herr T., Nashed M., Joseph J., Perkins W.R., DiPetrillo K. (2018). Amikacin Liposome Inhalation Suspension (ALIS) Penetrates Non-tuberculous Mycobacterial Biofilms and Enhances Amikacin Uptake Into Macrophages. Front. Microbiol..

[102] Sarmah S., Baidya S., De M. (2025). Recent Advances in Lipid Nanoparticles: Nucleic Acid Therapeutics and Targeting Strategies. Small.

[103] Bhardwaj H., Jangde R.K. (2023). Current updated review on preparation of polymeric nanoparticles for drug delivery and biomedical applications. Next Nanotechnol..

[104] Municoy S., Álvarez Echazú M.I., Antezana P.E., Galdopórpora J.M., Olivetti C., Mebert A.M., Foglia M.L., Tuttolomondo M.V., Alvarez G.S., Hardy J.G. (2020). Stimuli-Responsive Materials for Tissue Engineering and Drug Delivery. Int. J. Mol. Sci..

[105] van den Berg A.I.S., Yun C.-O., Schiffelers R.M., Hennink W.E. (2021). Polymeric delivery systems for nucleic acid therapeutics: Approaching the clinic. J. Control. Release.

[106] Wang H., Yuan Y., Qin L., Yue M., Xue J., Cui Z., Zhan X., Gai J., Zhang X., Guan J. (2024). Tunable rigidity of PLGA shell-lipid core nanoparticles for enhanced pulmonary siRNA delivery in 2D and 3D lung cancer cell models. J. Control. Release.

[107] Shen A.M., Minko T. (2020). Pharmacokinetics of inhaled nanotherapeutics for pulmonary delivery. J. Control. Release Off. J. Control. Release Soc..

[108] Pandey S., Shaikh F., Gupta A., Tripathi P., Yadav J.S. (2022). A Recent Update: Solid Lipid Nanoparticles for Effective Drug Delivery. Adv. Pharm. Bull..

[109] Costabile G., Conte G., Brusco S., Savadi P., Miro A., Quaglia F., d’Angelo I., Ungaro F. (2024). State-of-the-Art Review on Inhalable Lipid and Polymer Nanocarriers: Design and Development Perspectives. Pharmaceutics.

[110] Abu Elella M.H., Al Khatib A.O., Al-Obaidi H. (2024). Spray-Dried Nanolipid Powders for Pulmonary Drug Delivery: A Comprehensive Mini Review. Pharmaceutics.

[111] Wang W., Zhu R., Xie Q., Li A., Xiao Y., Li K., Liu H., Cui D., Chen Y., Wang S. (2012). Enhanced bioavailability and efficiency of curcumin for the treatment of asthma by its formulation in solid lipid nanoparticles. Int. J. Nanomed..

[112] Vishwakarma K., Handa P., Tawfeeq W.S., Hassan M.A. (2026). Recent advancements in drug nanocrystals: Innovation in formulation and drug delivery. OpenNano.

[113] Khan S., Sharma A., Jain V. (2023). An Overview of Nanostructured Lipid Carriers and its Application in Drug Delivery through Different Routes. Adv. Pharm. Bull..

[114] Knap K., Kwiecień K., Reczyńska-Kolman K., Pamuła E. (2023). Inhalable microparticles as drug delivery systems to the lungs in a dry powder formulations. Regen. Biomater..

[115] Rajoriya V., Gupta R., Vengurlekar S., Surendra Singh U. (2024). Nanostructured lipid carriers (NLCs): A promising candidate for lung cancer targeting. Int. J. Pharm..

[116] Zhang X., Chan H.W., Shao Z., Wang Q., Chow S., Chow S.F. (2025). Navigating translational research in nanomedicine: A strategic guide to formulation and manufacturing. Int. J. Pharm..

[117] Berikkhanova K., Inuwa I., Jibo A.G., Berikkhanov N., Bikhanov N., Sultan Y., Omarbekov A. (2025). Hybrid Nanocarriers for Cancer Therapy: Advancements in Co-Delivery of Gene Therapy and Immunotherapy. Int. J. Mol. Sci..

[118] Kassaee S.N., Richard D., Ayoko G.A., Islam N. (2024). Lipid polymer hybrid nanoparticles against lung cancer and their application as inhalable formulation. Nanomedicine.

[119] Jacob S., Varkey N.R., Boddu S.H.S., Gorain B., Rao R., Nair A.B. (2025). Advances in Lipid-Polymer Hybrid Nanoparticles: Design Strategies, Functionalization, Oncological and Non-Oncological Clinical Prospects. Pharmaceuticals.

[120] Zheng J., Sun Y., Shen Y., Zhou Z. (2025). Surface engineering of nanoparticles for precision medicine. Precis. Med. Eng..

[121] Ghazal H., Waqar A., Yaseen F., Shahid M., Sultana M., Tariq M., Bashir M.K., Tahseen H., Raza T., Ahmad F. (2024). Role of nanoparticles in enhancing chemotherapy efficacy for cancer treatment. Next Mater..

[122] Leong E.W.X., Ge R. (2022). Lipid Nanoparticles as Delivery Vehicles for Inhaled Therapeutics. Biomedicines.

[123] Panovich P.M., Ganesan A., Angadi A.R., Piotrowski-Daspit A.S. (2026). Recent advances in the application of polymeric nanoparticles to the pulmonary delivery of mRNA. Nanomedicine.

[124] Mondal S.K., Chakraborty S., Manna S., Mandal S.M. (2024). Antimicrobial nanoparticles: Current landscape and future challenges. RSC Pharm..

[125] Rowe S.M., Zuckerman J.B., Dorgan D., Lascano J., McCoy K., Jain M., Schechter M.S., Lommatzsch S., Indihar V., Lechtzin N. (2023). Inhaled mRNA therapy for treatment of cystic fibrosis: Interim results of a randomized, double-blind, placebo-controlled phase 1/2 clinical study. J. Cyst. Fibros..

[126] De Soyza A., Aksamit T., Bandel T.-J., Criollo M., Elborn J.S., Operschall E., Polverino E., Roth K., Winthrop K.L., Wilson R. (2018). RESPIRE 1: A phase III placebo-controlled randomised trial of ciprofloxacin dry powder for inhalation in non-cystic fibrosis bronchiectasis. Eur. Respir. J..

[127] Haworth C.S., Bilton D., Chalmers J.D., Davis A.M., Froehlich J., Gonda I., Thompson B., Wanner A., O’Donnell A.E. (2019). Inhaled liposomal ciprofloxacin in patients with non-cystic fibrosis bronchiectasis and chronic lung infection with *Pseudomonas aeruginosa* (ORBIT-3 and ORBIT-4): Two phase 3, randomised controlled trials. Lancet Respir. Med..

[128] Khan O., Chaudary N. (2020). The Use of Amikacin Liposome Inhalation Suspension (Arikayce) in the Treatment of Refractory Nontuberculous Mycobacterial Lung Disease in Adults. Drug Des. Dev. Ther..

[129] VanDevanter D.R., Geller D.E. (2011). Tobramycin administered by the TOBI® Podhaler® for persons with cystic fibrosis: A review. Med. Devices Evid. Res..

[130] Ramanathan R., Rasmussen M.R., Gerstmann D.R., Finer N., Sekar K. (2004). A randomized, multicenter masked comparison trial of poractant alfa (Curosurf) versus beractant (Survanta) in the treatment of respiratory distress syndrome in preterm infants. Am. J. Perinatol..

[131] Bui V.K., Moon J.-Y., Chae M., Park D., Lee Y.-C. (2020). Prediction of Aerosol Deposition in the Human Respiratory Tract via Computational Models: A Review with Recent Updates. Atmosphere.

[132] Vora L.K., Gholap A.D., Jetha K., Thakur R.R., Solanki H.K., Chavda V.P. (2023). Artificial Intelligence in Pharmaceutical Technology and Drug Delivery Design. Pharmaceutics.

[133] Serrano D.R., Luciano F.C., Anaya B.J., Ongoren B., Kara A., Molina G., Ramirez B.I., Sánchez-Guirales S.A., Simon J.A., Tomietto G. (2024). Artificial Intelligence (AI) Applications in Drug Discovery and Drug Delivery: Revolutionizing Personalized Medicine. Pharmaceutics.

[134] Shiammala P.N., Duraimutharasan N.K.B., Vaseeharan B., Alothaim A.S., Al-Malki E.S., Snekaa B., Safi S.Z., Singh S.K., Velmurugan D., Selvaraj C. (2023). Exploring the artificial intelligence and machine learning models in the context of drug design difficulties and future potential for the pharmaceutical sectors. Methods.

[135] Yadav A.P., Singh G., Singh M.K., Chaudhary A. (2025). Artificial intelligence in optimizing formulations and excipients: Revolutionizing pharmaceutical product development. J. Adv. Sci. Res..

[136] Dangeti A., Bynagari D.G., Vydani K. (2023). Revolutionizing Drug Formulation: Harnessing Artificial Intelligence and Machine Learning for Enhanced Stability, Formulation Optimization, and Accelerated Development. Int. J. Pharm. Sci. Med..

[137] Ali K.A., Mohin S.K., Mondal P., Goswami S., Ghosh S., Choudhuri S. (2024). Influence of artificial intelligence in modern pharmaceutical formulation and drug development. Future J. Pharm. Sci..

[138] Wang S., Di J., Wang D., Dai X., Hua Y., Gao X., Zheng A., Gao J. (2022). State-of-the-Art Review of Artificial Neural Networks to Predict, Characterize and Optimize Pharmaceutical Formulation. Pharmaceutics.

[139] Lin H.-L., Chiu Y.-W., Wang C.-C., Tung C.-W. (2022). Computational prediction of Calu-3-based in vitro pulmonary permeability of chemicals. Regul. Toxicol. Pharmacol..

[140] Goel K., Singh T.G., Paudel K.R., MacLoughlin R., Islam M.S., Kulkarni M.P., Dua K., Mujwar S. (2026). Molecular modelling–driven formulation design for targeted pulmonary drug delivery. Int. J. Pharm..

[141] Luo H., Yin W., Wang J., Zhang G., Liang W., Luo J., Yan C. (2024). Drug-drug interactions prediction based on deep learning and knowledge graph: A review. iScience.

[142] Zirkle J., Han X., Racz R., Samieegohar M., Chaturbedi A., Mann J., Chakravartula S., Li Z. (2023). Deep learning-enabled natural language processing to identify directional pharmacokinetic drug-drug interactions. BMC Bioinform..

[143] Jarallah S.J., Almughem F.A., Alhumaid N.K., Fayez N.A.L., Alradwan I., Alsulami K.A., Tawfik E.A., Alshehri A.A. (2025). Artificial intelligence revolution in drug discovery: A paradigm shift in pharmaceutical innovation. Int. J. Pharm..

[144] Doub W.H., Suman J.M., Copley M., Goodey A.P., Hosseini S., Mitchell J.P. (2023). Laboratory Performance Testing of Aqueous Nasal Inhalation Products for Droplet/Particle Size Distribution: An Assessment from the International Pharmaceutical Aerosol Consortium on Regulation and Science (IPAC-RS). AAPS PharmSciTech.

[145] Sadafi H., Monshi Tousi N., De Backer W., De Backer J. (2024). Validation of computational fluid dynamics models for airway deposition with SPECT data of the same population. Sci. Rep..

[146] Labiris N.R., Dolovich M.B. (2003). Pulmonary drug delivery. Part II: The role of inhalant delivery devices and drug formulations in therapeutic effectiveness of aerosolized medications. Br. J. Clin. Pharmacol..

[147] Tian G., Hindle M., Lee S., Longest P.W. (2015). Validating CFD Predictions of Pharmaceutical Aerosol Deposition with In Vivo Data. Pharm. Res..

[148] Ladumor M.K., Unadkat J.D. (2022). Predicting Regional Respiratory Tissue and Systemic Concentrations of Orally Inhaled Drugs through a Novel PBPK Model. Drug Metab. Dispos. Biol. Fate Chem..

[149] Deepika D., Kumar V. (2023). The Role of “Physiologically Based Pharmacokinetic Model (PBPK)” New Approach Methodology (NAM) in Pharmaceuticals and Environmental Chemical Risk Assessment. Int. J. Environ. Res. Public Health.

[150] Vulović A., Šušteršič T., Cvijić S., Ibrić S., Filipović N. (2018). Coupled in silico platform: Computational fluid dynamics (CFD) and physiologically-based pharmacokinetic (PBPK) modelling. Eur. J. Pharm. Sci. Off. J. Eur. Fed. Pharm. Sci..

[151] Khoa N.D., Kuga K., Ito K. (2025). Comprehensive integration framework of CFD—Local and whole body hybrid PBPK in indoor chemical exposure modeling: An inhalation exposure study. Sustain. Cities Soc..

[152] Gonsard A., Genet M., Drummond D. (2024). Digital twins for chronic lung diseases. Eur. Respir. Rev. Off. J. Eur. Respir. Soc..

[153] Feng Y., Chen X., Zhao J. (2018). Create the Individualized Digital Twin for Noninvasive Precise Pulmonary Healthcare. Significances Bioeng. Biosci..

[154] Nadeem M., Kostic S., Dornhöfer M., Weber C., Fathi M. (2025). A comprehensive review of digital twin in healthcare in the scope of simulative health-monitoring. Digit. Health.

[155] Gherman I.M., Abdallah Z.S., Pang W., Gorochowski T.E., Grierson C.S., Marucci L. (2023). Bridging the gap between mechanistic biological models and machine learning surrogates. PLoS Comput. Biol..

[156] Xiong S., Chen W., Jia X., Jia Y., Liu C. (2023). Machine learning for prediction of asthma exacerbations among asthmatic patients: A systematic review and meta-analysis. BMC Pulm. Med..

[157] McHeick H., Achouh P., Msheik B. (2025). Enhancing Healthcare with Digital Twins: A Comparative Approach Using AI and AI-Enhanced Digital Twins. Procedia Comput. Sci..

[158] Jiang J., Zhou Z., Peng T., Huang Z., Pan X., Wu C. (2026). Applications of AI/ML in accelerating the development of pulmonary drug delivery system. Acta Pharm. Sin. B.

[159] Khemasuwan D., Sorensen J.S., Colt H.G. (2020). Artificial intelligence in pulmonary medicine: Computer vision, predictive model and COVID-19. Eur. Respir. Rev. Off. J. Eur. Respir. Soc..

[160] Sunjaya A., Edwards G.D., Harvey J., Sylvester K., Purvis J., Rutter M., Shakespeare J., Moore V., El-Emir E., Doe G. (2025). Validation of artificial intelligence spirometry diagnostic support software in primary care: A blinded diagnostic accuracy study. ERJ Open Res..

[161] Al-Anazi S., Al-Omari A., Alanazi S., Marar A., Asad M., Alawaji F., Alwateid S. (2024). Artificial intelligence in respiratory care: Current scenario and future perspective. Ann. Thorac. Med..

[162] de Hond A.A.H., Shah V.B., Kant I.M.J., Van Calster B., Steyerberg E.W., Hernandez-Boussard T. (2023). Perspectives on validation of clinical predictive algorithms. npj Digit. Med..

[163] Cao B., Greiner R., Greenshaw A., Sui J. (2025). AI and Machine Learning Terminology in Medicine, Psychology, and Social Sciences: Tutorial and Practical Recommendations. J. Med. Internet Res..

[164] Gupta P., Pearce A.K., Pham T., Miller M., Brunetti K., Heskett K., Malhotra A., Mayampurath A., Afshar M. (2025). Artificial intelligence-driven decision support for patients with acute respiratory failure: A scoping review. Intensive Care Med. Exp..

[165] Woo M., Zhang L., Brown-Mulry B., Hwang I., Gichoya J.W., Gastounioti A., Banerjee I., Seyyed-Kalantari L., Trivedi H. (2025). Subgroup evaluation to understand performance gaps in deep learning-based classification of regions of interest on mammography. PLoS Digit. Health.

[166] Petrick N., Chen W., Delfino J.G., Gallas B.D., Kang Y., Krainak D., Sahiner B., Samala R.K. (2023). Regulatory considerations for medical imaging AI/ML devices in the United States: Concepts and challenges. J. Med. Imaging.

[167] Muehlematter U.J., Bluethgen C., Vokinger K.N. (2023). FDA-cleared artificial intelligence and machine learning-based medical devices and their 510(k) predicate networks. Lancet Digit. Health.

[168] Ahmed R., Tewes F., Aucamp M., Dube A. (2025). Formulation and clinical translation of inhalable nanomedicines for the treatment and prevention of pulmonary infectious diseases. Drug Deliv. Transl. Res..

[169] Lee J.W., Skibba M., Tang T., Noh H., Brasier A.R., Hong S. (2026). Inhalation-Based Nanoparticle Drug Delivery Targeting the Diseased Lower Airways in Idiopathic Pulmonary Fibrosis. Pharmaceutics.

[170] Öztürk K., Kaplan M., Çalış S. (2024). Effects of nanoparticle size, shape, and zeta potential on drug delivery. Int. J. Pharm..

[171] Gorohovs M., Dekhtyar Y. (2025). Surface Functionalization of Nanoparticles for Enhanced Electrostatic Adsorption of Biomolecules. Molecules.

[172] Liu Y.-Y., Sun Y.-Y., Guo Y., Chen L.-L., Guo J.-H., Wang H. (2025). Surface Charge Affects the Intracellular Fate and Clearance Dynamics of CdSe/ZnS Quantum Dots in Macrophages. Nanomaterials.

[173] Pielenhofer J., Meiser S.L., Gogoll K., Ciciliani A.-M., Denny M., Klak M., Lang B.M., Staubach P., Grabbe S., Schild H. (2023). Quality by Design (QbD) Approach for a Nanoparticulate Imiquimod Formulation as an Investigational Medicinal Product. Pharmaceutics.

[174] Gurba-Bryśkiewicz L., Maruszak W., Smuga D.A., Dubiel K., Wieczorek M. (2023). Quality by Design (QbD) and Design of Experiments (DOE) as a Strategy for Tuning Lipid Nanoparticle Formulations for RNA Delivery. Biomedicines.

[175] Mohseni-Motlagh S.F., Dolatabadi R., Baniassadi M., Baghani M. (2023). Application of the Quality by Design Concept (QbD) in the Development of Hydrogel-Based Drug Delivery Systems. Polymers.

[176] Lin K.-C., Lin H.-Y., Yang C.-Y., Chu Y.-L., Xie R.-H., Wang C.-M., Tseng Y.-L., Chen H.R., Chung J.H.Y., Yang J.-W. (2025). Inhalable Mucociliary-On-Chip System Revealing Pulmonary Clearance Dynamics in Nanodrug Delivery. ACS Nano.

[177] Patel B., Gupta N., Ahsan F. (2024). Barriers that Inhaled Particles Encounter. J. Aerosol Med. Pulm. Drug Deliv..

[178] Peivandi Z., Shirazi F.H., Teimourian S., Farnam G., Babaei V., Mehrparvar N., Koohsari N., Ashtarinezhad A. (2024). Silica nanoparticles-induced cytotoxicity and genotoxicity in A549 cell lines. Sci. Rep..

[179] Michelini S., Mawas S., Kurešepi E., Barbero F., Šimunović K., Miremont D., Devineau S., Schicht M., Ganin V., Haugen Ø.P. (2025). Pulmonary hazards of nanoplastic particles: A study using polystyrene in in vitro models of the alveolar and bronchial epithelium. J. Nanobiotechnol..

[180] Costa C., Padrela L. (2025). Progress on drug nanoparticle manufacturing: Exploring the adaptability of batch bottom-up approaches to continuous manufacturing. J. Drug Deliv. Sci. Technol..

[181] Cojocaru E., Petriș O.R., Cojocaru C. (2024). Nanoparticle-Based Drug Delivery Systems in Inhaled Therapy: Improving Respiratory Medicine. Pharmaceuticals.

[182] Van Norman G.A. (2019). Limitations of Animal Studies for Predicting Toxicity in Clinical Trials: Is it Time to Rethink Our Current Approach?. JACC Basic Transl. Sci..

[183] Mossadeq S., Shah R., Shah V., Bagul M. (2022). Formulation, Device, and Clinical Factors Influencing the Targeted Delivery of COVID-19 Vaccines to the Lungs. AAPS PharmSciTech.

[184] Korde A., Mikolajczak R., Kolenc P., Bouziotis P., Westin H., Lauritzen M., Koole M., Herth M.M., Bardiès M., Martins A.F. (2022). Practical considerations for navigating the regulatory landscape of non-clinical studies for clinical translation of radiopharmaceuticals. EJNMMI Radiopharm. Chem..

[185] Hua S., de Matos M.B.C., Metselaar J.M., Storm G. (2018). Current Trends and Challenges in the Clinical Translation of Nanoparticulate Nanomedicines: Pathways for Translational Development and Commercialization. Front. Pharmacol..

